# Stat5 induces androgen receptor (*AR*) gene transcription in prostate cancer and offers a druggable pathway to target AR signaling

**DOI:** 10.1126/sciadv.adi2742

**Published:** 2024-02-28

**Authors:** Cristina Maranto, Lavannya Sabharwal, Vindhya Udhane, Samuel P. Pitzen, Braedan McCluskey, Songyan Qi, Christine O’Connor, Savita Devi, Scott Johnson, Kenneth Jacobsohn, Anjishnu Banerjee, Kenneth A. Iczkowski, Liang Wang, Scott M. Dehm, Marja T. Nevalainen

**Affiliations:** ^1^ Department of Pathology, Medical College of Wisconsin, Milwaukee, WI 53226, USA.; ^2^ Masonic Cancer Center, University of Minnesota, Minneapolis, MN 55455, USA.; ^3^ Graduate Program in Molecular, Cellular, and Developmental Biology and Genetics, University of Minnesota, Minneapolis, MN 55455, USA.; ^4^ Minnesota Supercomputing Institute, University of Minnesota, Minneapolis, MN 55455, USA.; ^5^ Graduate Program in Biochemistry, Molecular Biology and Biophysics, University of Minnesota, Minneapolis, MN 55455, USA.; ^6^ Department of Urology, Medical College of Wisconsin, Milwaukee, WI 53226, USA.; ^7^ Institute for Health and Equity, Medical College of Wisconsin, Milwaukee, WI 53226, USA.; ^8^ Department of Tumor Biology, Moffitt Cancer Center, 12902 USF Magnolia Drive, Tampa, FL 33612, USA.; ^9^ Department of Laboratory Medicine and Pathology, University of Minnesota, Minneapolis, MN 55455, USA.; ^10^ Department of Urology, University of Minnesota, Minneapolis, MN 55455, USA.; ^11^ Department of Pharmacology, Physiology and Cancer Biology, Sidney Kimmel Cancer Center at Jefferson Health, Thomas Jefferson University, Philadelphia, PA 19107, USA.

## Abstract

Androgen receptor (AR) drives prostate cancer (PC) growth and progression, and targeting AR signaling is the mainstay of pharmacological therapies for PC. Resistance develops relatively fast as a result of refueled AR activity. A major gap in the field is the lack of understanding of targetable mechanisms that induce persistent AR expression in castrate-resistant PC (CRPC). This study uncovers an unexpected function of active Stat5 signaling, a known promoter of PC growth and clinical progression, as a potent inducer of *AR* gene transcription. Stat5 suppression inhibited *AR* gene transcription in preclinical PC models and reduced the levels of wild-type, mutated, and truncated AR proteins. Pharmacological Stat5 inhibition by a specific small-molecule Stat5 inhibitor down-regulated Stat5-inducible genes as well as AR and AR-regulated genes and suppressed PC growth. This work introduces the concept of Stat5 as an inducer of *AR* gene transcription in PC. Pharmacological Stat5 inhibitors may represent a new strategy for suppressing AR and CRPC growth.

## Teaser

Pharmacological Stat5 inhibition provides a strategy to suppress androgen receptor gene transcription and prostate cancer growth.

## INTRODUCTION

The main protein that drives prostate cancer (PC) growth and progression is the androgen receptor (AR), a transcription factor induced by androgenic steroids (e.g., testosterone) to regulate the genetic network supporting PC growth (*1*). Therefore, targeting AR signaling by androgen deprivation therapy (ADT) is a mainstay for pharmacological treatment of men diagnosed with PC at an advanced stage or experiencing tumor recurrence after surgery (*2*, *3*). Furthermore, ADT is often included as an adjuvant therapy concurrently with radiation therapy for patients with unfavorable intermediate- or high-risk PC (*4*).

ADT can be conducted by suppressing circulating androgen levels with luteinizing hormone (LH) secretion inhibitors, by inhibiting androgen synthesis with CYP17A1 inhibitors such as abiraterone, and/or by inhibiting the AR directly with high-affinity antagonists such as enzalutamide (ENZ), apalutamide, and darolutamide (*2*, *3*, *5*–*9*). Over time, ADT leads to the emergence of lethal castrate-resistant PC (CRPC). CRPC is consistently caused by an acquired ability of tumors to reactivate AR through multiple mechanisms (*10*, *11*). In 60 to 65% of CRPC, amplification of the *AR* gene body and/or upstream enhancer occurs, which increases the levels of AR expression (*12*–*15*), including AR splice variants AR-V7 and AR-V9 that lack the ligand binding domain and display constitutive transcriptional activity (*16*–*23*). In 25 to 35% of CRPC, structural rearrangements alter the architecture of *AR* exons, which can promote the synthesis of diverse AR variant (AR-V) species such as AR-V12/ARv567es (*23*, *24*). Somatic *AR* point mutations occur in approximately 10 to 15% of CRPC, many of which broaden the repertoire of ligands that can activate the AR ligand binding domain (*12*, *13*). To bypass the adaptive changes occurring in the *AR* gene permitting AR-driven PC growth during ADT, therapeutic strategies that suppress the *AR* gene transcription in PC have the potential to provide a more prolonged clinical response by reducing the levels of these diverse truncated or mutated AR protein species.

Stat5 comprises two highly homologous isoforms Stat5a (94 kDa) and Stat5b (92 kDa) (referred to as Stat5), which are nucleocytoplasmic proteins acting both as cytoplasmic signaling proteins and nuclear transcription factors (*25*–*30*). Upon tyrosine phosphorylation by Jak2, Stat5 forms functional dimers that translocate to the nucleus, bind to specific Stat5 DNA response elements, and activate transcription (*27*). The transcriptional program regulated by Stat5 sustains PC cell viability and CRPC growth (*31*–*40*). Blocking Stat5 signaling induces apoptotic death of PC cells and suppresses the growth of both androgen-sensitive and CRPC tumors, and patient-derived PCs cultured ex vivo (*32*, *34*–*36*, *38*, *40*–*42*). Conversely, overexpression of active Stat5 induces viability of PC cells in vitro, growth of PC tumors in mice (*43*), and confers resistance to ENZ in vitro and in vivo (*40*). As indicative of the Stat5 involvement in clinical PC progression, Stat5 activation in clinical PC predicts early disease recurrence and PC-specific death in patients (*44*–*46*). We have developed a Stat5 inhibitor, IST5-002 (*36*, *39*, *40*, *47*), which binds to the SH2-domain of Stat5 and disrupts the docking of Stat5 to the receptor-tyrosine kinase complex. This leads to inhibition of Stat5 phosphorylation and dimerization at nanomolar concentrations in cell-based assays (*36*, *39*).

In this study, our work discovers a previously unknown and unexpected role of activated Stat5 signaling as a robust inducer of *AR* gene transcription in PC. This finding opens a new avenue for therapeutic targeting of AR signaling and PC growth by pharmacological inhibition of Stat5. Down-regulation of Stat5 activity suppressed *AR* gene transcription in diverse cell lines and patient-derived models of PC and CRPC, thereby reducing the levels of wild-type, mutated, and truncated AR proteins. Stat5 inhibitor, IST5-002, at nanomolar concentrations suppresses AR levels and PC growth in experimental PC models, including CRPC cells, CRPC tumors, and patient-derived PCs cultured ex vivo in tumor explant cultures. In summary, this work introduces the concept that Stat5 is an inducer of the *AR* gene transcription in PC. Therefore, pharmacological inhibitors of activated Stat5 may represent a novel strategy for suppressing AR and the growth of PC.

## RESULTS

### Stat5 increases the expression of AR mRNA and protein in prostate cancer

**Fig. 1.**
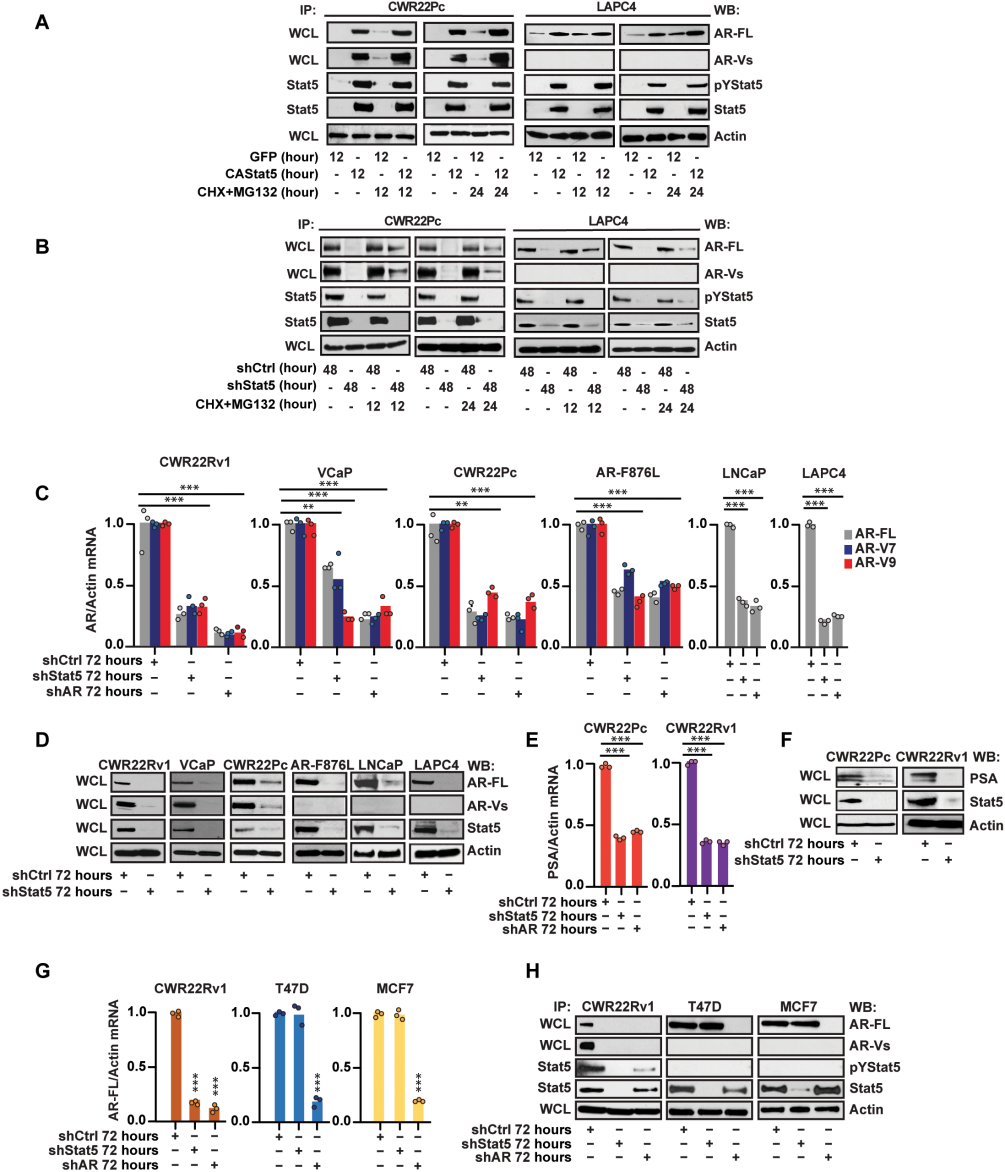
Active Stat5 increases protein levels of full-length androgen receptor (AR-FL) and AR variants (AR-Vs) through the induction of AR mRNA levels in PC. (**A**) CA Stat5a/b or green fluorescent protein (GFP) was lentivirally expressed in CWR22Pc and LAPC for 12 hours followed by CHX (30 μM) and MG132 (10 μM) for 12 or 24 hours. AR protein levels were determined by Western blotting (WB) of whole-cell lysates (WCLs) with actin as a loading control. Active Stat5 levels were determined by immunoprecipitation (IP) of Stat5 followed by WB for phosphorylated Stat5 (pStat5) and total Stat5 (A, **B**, **D**, **F**, and **H**). (B) Stat5 was suppressed by lentiviral shStat5 expression in CWR22Pc and LAPC4 cells for 48 hours followed by CHX (30 μM) and MG132 (10 μM) for 12 or 24 hours. (**C**) Lentiviral shStat5 expression with control (shCtrl) for 72 hours in CWR22Rv1, VCaP, CWR22Pc, and ENZ-resistant CWR22Pc-expressing AR-F876L, LNCaP, and LAPC4. AR-FL, AR-V7, and AR-V9 mRNA levels were determined by qRT-PCR with lentiviral shRNA depletion of AR (shAR) for 72 hours as control. (D) The same cell lines were transduced by lenti-shStat5 for 72 hours followed by the IP of AR-FL, AR-V, Stat5, and actin by IP and WB. (**E**) Lentiviral transduction of Stat5 shRNA for 72 hours in CWR22Pc and CWR22Rv1 cells in comparison to lenti-shCtrl or lenti-shAR followed by the determination of PSA mRNA and protein (F) levels by qRT-PCR and WB. AR was suppressed by the lentiviral expression of shAR as a control for qRT-PCR. (**G**) Lentiviral expression of shStat5 versus shCtrl or shAR for 72 hours in T47D, MCF7, and CWR22Rv1 cells followed by the determination of AR mRNA and protein (H) levels by qRT-PCR and WB using lenti-shAR as control. Data are represented by bars. Hypothesis tests were performed using ANOVA with post hoc testing using the Student’s *t* test, with Bonferroni multiplicity corrections. Significance levels indicated for relevant hypotheses as *P < 0.05, **P < 0.01, and ***P < 0.001.

We and others have previously shown that depletion of Stat5 in PC cells reduces the stability of the AR protein in PC cells due to increased flux of AR through the proteasome (*42*, *48*). This concept was further supported by an analysis of a cohort of 442 clinical PCs in which high AR protein expression correlated with high Stat5 protein levels (*48*). To investigate whether pharmacological inhibitors of Jak2-Stat5 signaling could be used to reduce AR protein stability in PC, we evaluated whether Stat5 prevention of the AR from proteasomal degradation is dependent on the activation status of Stat5. We overexpressed constitutively active (CA) Stat5a/b mutants (Stat5aS710F and Stat5bS715F) (*35*) (CAStat5) by lentivirus in CWR22Pc and LAPC4 cells followed by cotreatment of the cells with protein synthesis inhibitor cycloheximide (CHX) and proteasome inhibitor MG132 for 12 and 24 hours. Unexpectedly, while CAStat5 increased AR protein levels in PC cells, active Stat5 induction of the AR was not further improved by blockade of proteasomal degradation (Fig. 1A). These findings indicated that active Stat5 signaling is capable of inducing AR levels which, however, does not occur through stabilization of AR protein. At the same time, the knockdown of Stat5 by lentiviral transduction of shStat5a/b reduced AR protein levels, which was partially rescued by MG132 treatment and is consistent with the previously reported data (Fig. 1B) (*48*). Collectively, these results suggest that the presence of Stat5 protein, but not its activation, stabilizes AR protein in PC cells. The lentiviral transduction scheme is illustrated in fig. S1A, and the timelines chosen for CAStat5 overexpression versus Stat5 knockdown by shStat5 were supported by the preliminary studies shown in fig. S1 (B to D). To summarize, the data point to additional mechanisms being involved in Stat5-driven induction of AR levels in PC.

We next proceeded to evaluate whether Stat5 increases the expression of AR mRNA in PC. Stat5 was suppressed by the lentiviral expression of shStat5 with shAR as a control for AR knockdown. Stat5 depletion reduced mRNA levels of both the full-length AR (AR-FL) and AR variants AR-V7 and AR-V9 in PC cell lines positive for AR-FL and AR-Vs (CWR22Rv1, VCaP, CWR22Pc, and CWR22Pc line–expressing AR-F876) and in PC cells expressing exclusively the AR-FL (LNCaP and LAPC4) to a level achieved by lentiviral shAR (Fig. 1C and fig. S2). The suppression of AR-FL and AR-V mRNAs by Stat5 inhibition was reflected by decreased AR-FL and AR-V protein levels in PC cells (Fig. 1D and fig. S1). Simultaneously, the mRNA and protein levels of the AR-target gene prostate specific antigen (PSA) were suppressed by shStat5 (Fig. 1, E and F). While Stat5 knockdown down-regulated AR mRNA levels, depletion of AR by lentiviral shAR did not affect Stat5 mRNA levels in PC cells (fig. S2F).

Last, we sought to determine whether Stat5-regulation of AR mRNA occurs in other tissue types and evaluated two different AR-positive human breast cancer cell lines T47D and MCF-7. As shown in Fig. 1 (G and H), lentiviral shStat5 had no effect on either AR mRNA or protein levels in these non-PC cell lines. To conclude, these data indicate that Stat5 inhibition suppresses mRNA expression of AR-FL and AR-Vs in PC, which is reflected by reduced AR-FL and AR-V protein levels. In addition, the data suggest that Stat5 induction of the AR mRNA levels may be specific to PC cells.

### Active Stat5 induces AR-FL and AR-V mRNA levels in prostate cancer

**Fig. 2.**
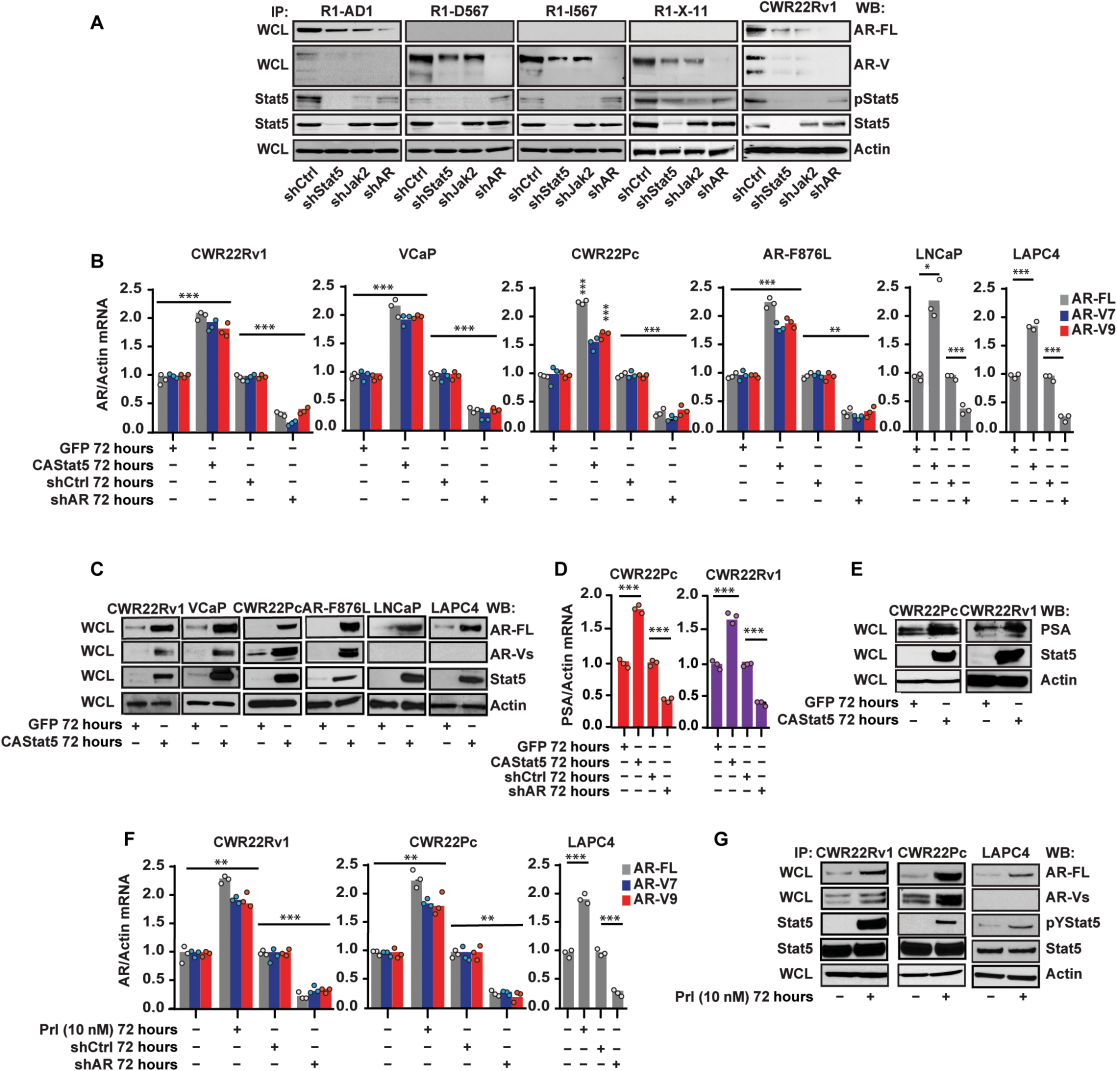
Activated Stat5 induces AR-FL and AR-V mRNA and protein expression in prostate cancer. (**A**) Genetically engineered human PC cell lines R1-AD1, R1-D567, R11567, R1-X-11, and CWR22Rv1 cells were transduced by lentivirus expressing shRNAs targeting Stat5, Jak2, and AR versus a scramble sequence (shCtrl) for 72 hours followed by the determination of AR-FL, AR-V, pStat5, and total Stat5 levels versus actin by IP and WB, as indicated. (**B**) CAStat5 or GFP was lentivirally expressed for 72 hours in CWR22Rv1, VCaP, CWR22Pc, and ENZ-resistant CWR22Pc-expressing AR-F876L, LNCaP, and LAPC4 cells. AR-FL, AR-V7, and AR-V9 mRNA levels were determined by qRT-PCR with shAR versus shCtrl for 72 hours as a control. (**C**) The same PC cell lines were transduced by lenti-CAStat5 for 72 hours followed by the determination of AR-FL, AR-V, Stat5, and actin by IP and WB, as indicated. (**D**) CAStat5 or GFP was transduced by lentivirus in CWR22Pc and CWR22Rv1 cells for 72 hours with a lentiviral expression of shCtrl or shAR as a comparison. PSA mRNA levels were determined by qRT-PCR and protein (**E**) levels by WB with actin as a loading control. (**F**) CWR22Rv1, CWR22Pc, and LAPC4 cells were treated with the cytokine prolactin (Prl) (10 nM) for 72 hours followed by the determination of AR-FL, AR-V7, and AR-V9 mRNA levels by qRT-PCR and (**G**) protein levels by WB. Activation of Stat5 was evaluated by the IP of Stat5 followed by WB of pStat5 and total Stat5. In parallel, AR was genetically suppressed by lentiviral expression of AR shRNA as a control for AR mRNA suppression and detection. Hypothesis tests were performed using ANOVA with post hoc testing using the Student’s *t* test with Bonferroni multiplicity corrections. Significance levels are indicated for relevant hypotheses as *P < 0.05, **P < 0.01, and ***P < 0.001.

We next tested whether Stat5 and its activation are required for the expression of AR-Vs arising from *AR* gene rearrangements occurring in clinical PCs (*23*, *24*). For this, we used R1-D567, R1-I567, and R1-X-11 cell lines that have been engineered to express AR-Vs but not AR-FL under the control of the endogenous *AR* locus (*24*, *49*). In these cell line models, the knockdown of Stat5 or Jak2 with lentiviral shRNA reduced the levels of AR-V proteins (Fig. 2A). Lentiviral expression of shRNAs targeting Stat5 and Jak2 shRNA reduced AR-FL levels in R1-AD1 cells, which is the parental line from which R1-D567, R1-I567, and R1-X-11 were derived. Similarly, shStat5 and shJak2 reduced AR-FL and AR-V levels in CWR22Rv1 cells, which harbor a 35-kb tandem duplication within the *AR* gene (Fig. 2A) (*49*). Together, these results suggest that Stat5 knockdown suppresses AR-V mRNA levels in the absence of the AR-FL in PC cell lines modeling genetic rearrangements occurring in the *AR* gene in clinical PCs. In addition, Jak2 knockdown by shJak2 decreased AR levels suggesting that the activation of Stat5 is required for Stat5 regulation of the AR. Next, we expressed CA Stat5a/b (*40*) versus green fluorescent protein (GFP) in PC cells for 72 hours using lentiviral gene transduction. CAStat5 induced mRNA levels of AR-FL, AR-V7, and AR-V9 in CWR22Rv1, VCaP, CWR22Pc, and in the ENZ-resistant line of CWR22Pc-expressing AR-F876L cells. In addition, CAStat5 induced AR-FL mRNA and protein levels in LNCaP and LAPC4 PC cells which do not express AR-Vs (Fig. 2B and fig. S2). The induction of AR-FL and AR-V mRNA by Stat5 corresponded with an increase in protein levels of AR-FL and AR-Vs (Fig. 2C and fig. S1). Furthermore, PSA mRNA and protein levels were increased in PC cells expressing CAStat5 (Fig. 2, D and E). To evaluate whether the activation of endogenous Stat5 is sufficient to induce AR-FL and AR-V mRNA, CWR22Rv1, we treated CWR22Pc and LAPC4 cells with the cytokine prolactin (Prl), which is known to activate the canonical Jak2-Stat5 pathway in PC cells (*35*, *50*). Similar to CAStat5, Prl increased mRNA and protein levels of AR-FL and AR-Vs in CWR22Rv1, CWR22Pc, and LAPC4 cells in tandem with inducing Stat5 activation (Fig. 2, F and G, and fig. S3). In summary, these data demonstrate that activated Stat5 increases AR-FL and AR-V mRNA and protein levels in PC cells.

### The Stat5 inhibitor IST5-002 suppresses AR-FL and AR-V mRNA levels in prostate cancer

**Fig. 3.**
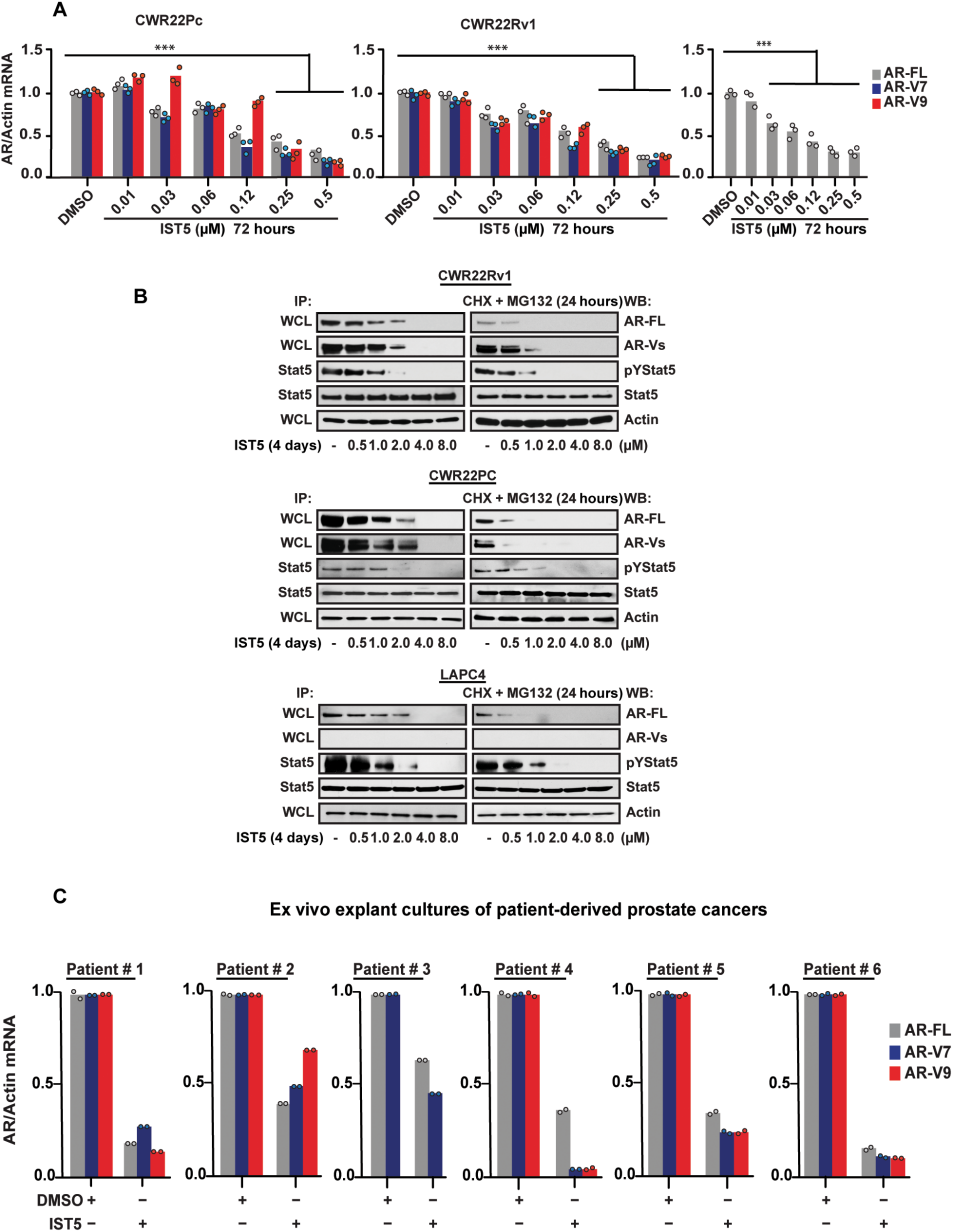
Stat5 inhibitor, IST5-002 (IST5), suppresses AR-FL and AR-V mRNA and protein levels in prostate cancer cells but does not destabilize AR. (**A**) CWR22Pc, CWR22Rv1, and LAPC cells were treated with increasing concentrations of IST5-002 with DMSO (vehicle) as a control for 72 hours followed by the determination of AR mRNA levels by qRT-PCR. (**B**) IST5-002 suppresses the protein levels of AR-FL and AR-Vs but not the stability of the AR protein in PC cells. CWR22Rv1, CWR22Pc, and LAPC4 cells were treated for 96 hours with increasing concentrations of IST5-002. The cells were treated with CHX (30 μM) and MG132 (10 μM) for 24 hours, followed by the determination of the levels of the AR protein by WB of WCLs with actin blotting as a loading control. The levels of Stat5 activation were determined by the IP of Stat5 followed by WB for pStat5 and total Stat5. (**C**) Six localized PCs obtained from radical prostatectomies were cultured ex vivo in tumor explant cultures in the presence of vehicle (DMSO) or IST5-002 for 7 days followed by the determination of AR-FL, AR-V7, and AR-V9 mRNA levels by qRT-PCR. Hypothesis tests were performed using ANOVA with post hoc testing using the Student’s *t* test with Bonferroni multiplicity corrections. Repeated measures adjustments were included for (C). Significance levels are indicated for relevant hypotheses as *P < 0.05, **P < 0.01, and ***P < 0.001.

Having established that activated Stat5 up-regulates AR levels in PC by induction of AR-FL and AR-V mRNA, we investigated if pharmacological inhibition of Stat5 activity can be used to suppress AR mRNA levels in PC. We previously identified (*36*) and characterized (*39*) a specific small-molecule Stat5 inhibitor, IST5-002, which docks to the Stat5 SH2 domain and obstructs both phosphorylation and dimerization of Stat5 (*36*, *39*). Treatment of CWR22Pc, CWR22Rv1, and LAPC4 cells with increasing concentrations of IST5-002 for 72 hours resulted in a dose-dependent decrease in AR-FL and AR-V mRNA levels with IC50s ranging from 120 to 250 nM (Fig. 3A). IST5-002 suppression of AR mRNA levels was accompanied by decreases in AR-FL and AR-V protein levels (Fig. 3B). To further verify the mechanism of action of IST5-002 on reducing AR in PC, CWR22Rv1, CWR22PC, and LAPC4 cells were treated with increasing concentrations of IST5-002 for 72 hours followed by blockade of protein synthesis and proteasomal degradation for 24 hours. Inhibition of the proteasome by MG132 did not rescue the loss of AR protein levels in cells treated by IST5-002 (Fig. 3B). In conclusion, these data indicate that pharmacological inhibition of Stat5 activity by IST5-002 suppresses AR mRNA and protein levels but does not de-stabilize AR-FL and AR-V proteins in PC cells. To evaluate whether Stat5 signaling is critical for AR mRNA expression in clinical patient-derived PCs, we used an ex vivo 3D tumor explant culture system of patient-derived PCs which we have established and characterized previously (*35*, *36*, *47*, *51*, *52*). PCs from six individual patients (table S2) were cultured ex vivo in tumor explant cultures with IST5-002 or vehicle as a control. PCs from all six patients responded to the IST5-002 treatment with a 50 to 90% decrease in the mRNA levels encoding AR-FL and AR-Vs (Fig. 3C).

**Fig. 4.**
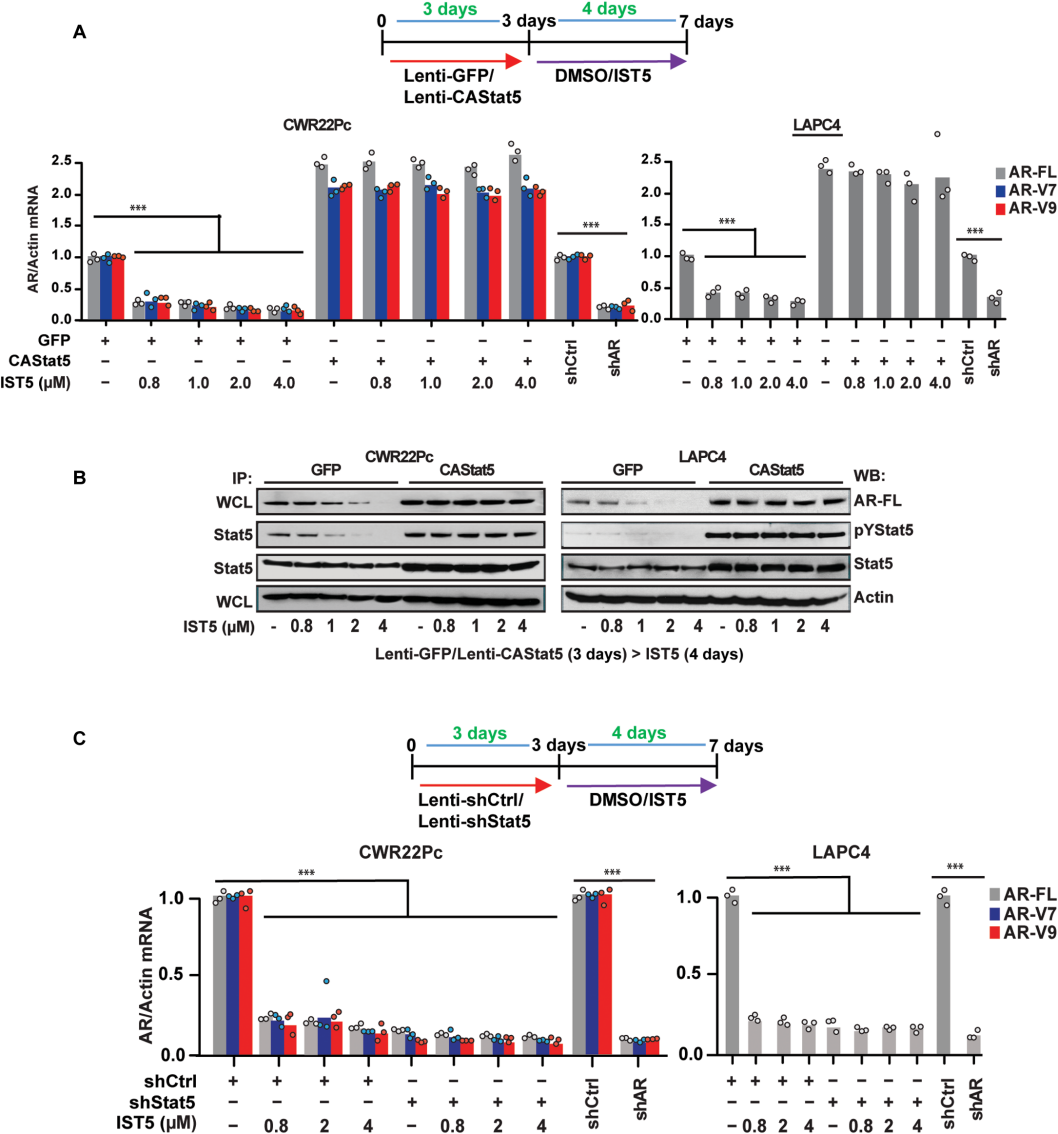
Stat5 inhibitor, IST5-002 (IST5), suppresses AR-FL and AR-V mRNA and protein levels via Stat5 in prostate cancer. (**A**) CA Stat5 or GFP was transduced to CWR22Pc and LAPC4 cells using lentivirus for 3 days before treatment of the cells with increasing concentrations of IST5-002. AR-FL, AR-V7, and AR-V9 mRNA levels were determined by qRT-PCR with shAR for 72 hours as a comparison. (**B**) Protein levels of AR-FL and AR-Vs were determined by WB with actin as a loading control. Stat5 was immunoprecipitated from the same lysates and blotted with antibodies against pStat5 and total Stat5. (**C**) Stat5 was suppressed by the lentiviral expression of shStat5 in CWR22Pc and LAPC4 cells for 3 days followed by treatment of the cells with increasing concentrations of IST5-002. AR-FL, AR-V7, and AR-V9 mRNA levels were determined by qRT-PCR with shAR for 72 hours as a comparison. Significance levels are indicated for relevant hypotheses as *P < 0.05, **P < 0.01, and ***P < 0.001.

To investigate whether IST5-002 specifically targets AR via suppression of Stat5 in PC, CA CAStat5, which is not inhibited by IST5-002 because of activating mutations in the transactivation domain of Stat5, was overexpressed in CWR22Pc and LAPC4 cells using lentivirus with lenti-GFP as control before IST5-002 treatment (Fig. 4, A and B). CAStat5 counteracted IST5-002–mediated suppression of FL-AR and AR-V mRNA (Fig. 4A) and protein levels in PC cells (Fig. 4B). Next, we tested whether the knockdown of Stat5, the target of IST5-002, would prevent IST5-002 from causing a further decrease in AR mRNA and protein levels. In CWR22Pc and LAPC4 cells with Stat5 knockdown, IST5-002 treatment did not induce an additional decrease in AR mRNA levels, providing additional support that IST5-002 suppression of AR was mediated via Stat5 (Fig. 4C). Overall, these data establish that Stat5 induces AR-FL and AR-V mRNA expression in PC, which can be inhibited by IST5-002.

### Active Stat5 induces the transcription of the *AR* gene in prostate cancer

**Fig. 5.**
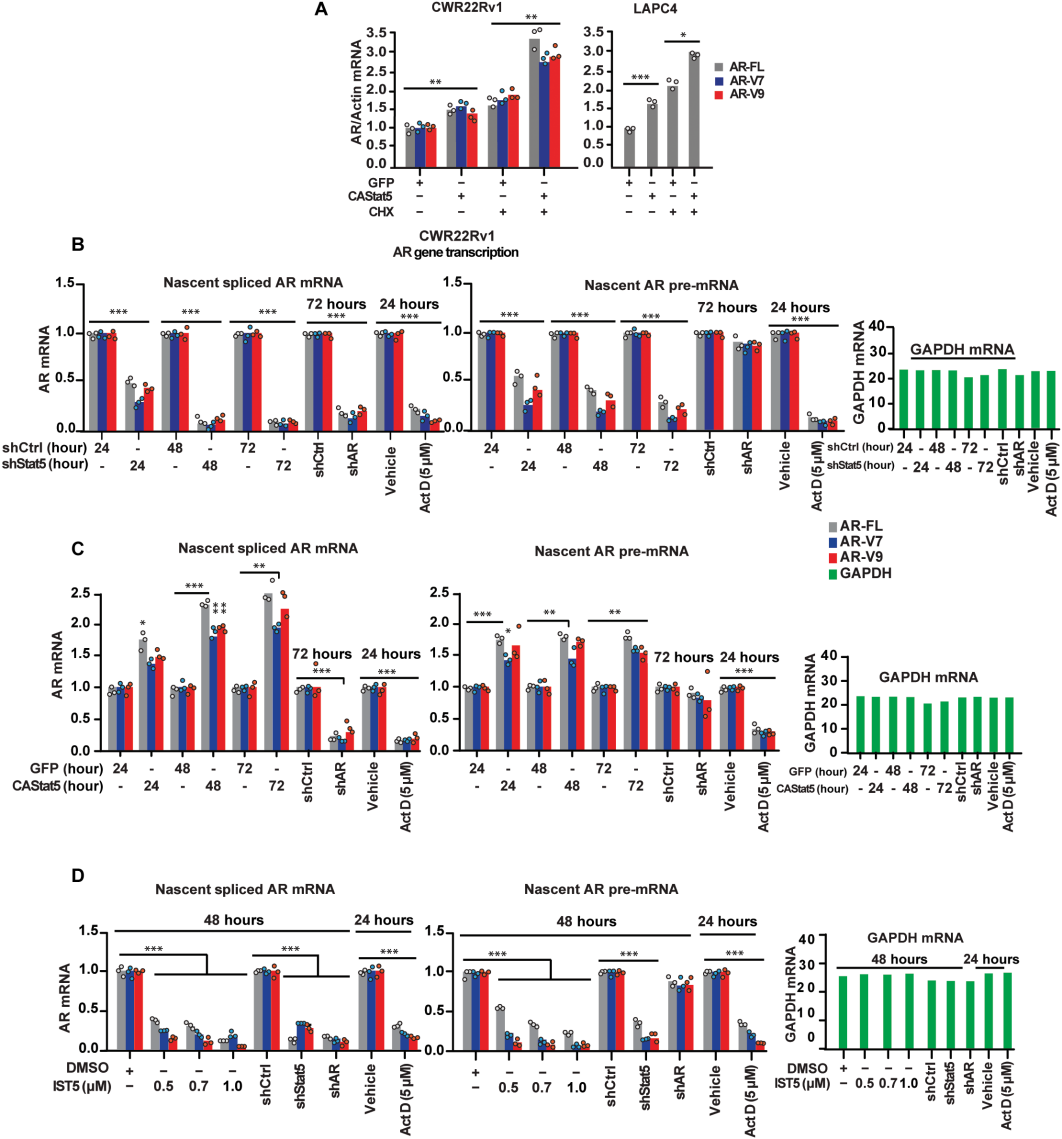
Active Stat5 induces transcription of the *AR* gene in prostate cancer. (**A**) CA Stat5 or GFP was lentivirally expressed in CWR22Pc and LAPC-4 cells for 6 hours followed by treatment with CHX (20 μM). AR-FL, AR-V7, and AR-V9 mRNA levels were determined by qRT-PCR. (**B**) CWR22Rv1 cells were transduced with lentivirus expressing Stat5 shRNA, ShCtrl, (**C**) CAStat5, and GFP for 24, 48, or 72 hours or (**D**) treated with IST5-002 (IST5) at increasing concentrations for 24 or 48 hours, as indicated. The nascent cellular mRNA was pulse-labeled with the ribonucleotide homolog ethylene uridine. Click-iT chemistry was used to biotinylate nascent transcripts, followed by streptavidin capture and quantification of spliced AR mRNA and nascent AR pre-mRNA levels by qRT-PCR. Control groups included shAR versus shCtrl for indicated time points or treatment of the cells with actinomycin D (ActD) (5 μM) for 24 hours. Glyceraldehyde-3-phosphate dehydrogenase (GAPDH) levels were determined in the same samples by qRT-PCR. Hypothesis tests were performed using ANOVA with post hoc testing using the Student’s *t* test with Bonferroni multiplicity corrections. Significance levels are indicated for relevant hypotheses as *P < 0.05, **P < 0.01, and ***P < 0.001.

Given that Stat5 increases AR mRNA levels in PC, we next tested whether this was a direct effect of active Stat5 or a secondary effect mediated by an intermediary Stat5-regulated factor. Lentiviral CAStat5 was expressed in CWR22Rv1 and LAPC4 cells for 6 hours (the lentiviral transduction scheme is presented in fig. S1A), followed by CHX treatment for 24 hours to block new protein synthesis. While CHX increased the basal AR mRNA levels, CHX treatment did not abrogate induction of AR mRNA levels by CAStat5 (Fig. 5A). These data suggest that CAStat5 does not increase AR levels through induction of expression of another protein. Next, we evaluated whether CAStat5 increases AR mRNA stability by treating CWR22Rv1 cells with actinomycin D to block transcription. The kinetics of AR mRNA decay following actinomycin D treatment were similar in cells expressing CAStat5 or GFP as a control, ruling out that CAStat5 improves AR mRNA stability (fig. S4). Collectively, these results implicated that Stat5 up-regulated *AR* gene transcription in PC cells. To further test this concept, we pulse-labeled nascent transcribed RNA in CWR22Rv1 cells expressing lentiviral Stat5 shRNA or CAStat5. As expected, the levels of nascent AR pre-mRNA and nascent spliced AR-FL and AR-V mRNAs were reduced by Stat5 knockdown (Fig. 5B) and increased by CAStat5 (Fig. 5C). Consistent with these results, IST5-002 reduced the levels of nascent AR pre-mRNA and nascent spliced AR-FL and AR-V mRNAs levels (Fig. 5D). Collectively, these data imply that Stat5 promotes the transcription of the *AR* gene in PC, which can be inhibited by nanomolar concentrations of the pharmacological Stat5 inhibitor, IST5-002. The data further suggest that active Stat5 induction of AR gene transcription is not mediated through the expression of an intermediary protein.

### The Stat5 transcriptome in PC cells includes *AR *and key AR target genes

**Fig. 6.**
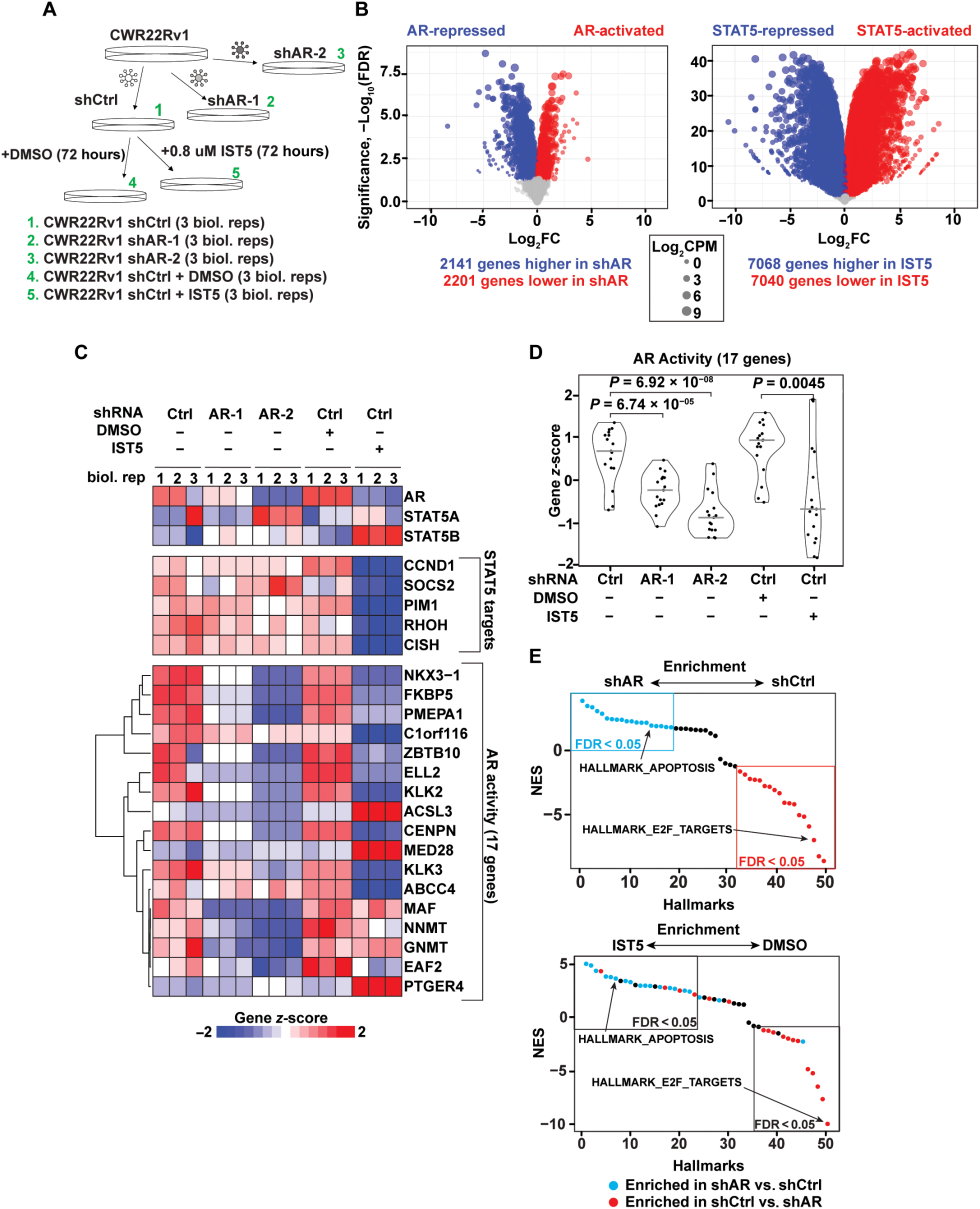
The Stat5 transcriptome in PC cells includes AR and key AR target genes. (**A**) Experimental design for comparing transcriptome-wide effects of AR knockdown with shRNA (shAR-1 and shAR-2) versus Stat5 inhibition with IST5-002 (IST5) in CWR22Rv1 cells using RNA-seq. (**B**) Volcano plots of differentially expressed genes in CWR22Rv1 cells infected with shAR or shCtrl lentivirus (left) or CWR22Rv1 cells infected with shCtrl lentivirus and treated with 0.8 μM IST5 or DMSO (right). Colored dots reflect genes exceeding cutoffs of false discovery rate (FDR) < 0.05 and fold change > 2. The size of each dot reflects the log_2_ of counts per million (cpm) measured for that gene. (**C**) Gene expression heatmap. Each row reflects the gene expression *z*-score for AR, STAT5A, STAT5B, 5 known STAT5 targets, and 17 AR target genes. (**D**) Violin plots comparing *z*-scores for each of the 17 AR target genes in (C) within indicated treatment groups. Gray lines indicate the median. *P* values are from the Student’s *t* test. (**E**) Normalized enrichment scores (NES) for the 50 HALLMARK gene sets derived from gene set enrichment analysis (GSEA) in the CWR22Rv1 RNA-seq data reflecting AR activity (shAR versus shCtrl, top) or STAT5 activity (IST5-002 versus DMSO, bottom). Dots are colored blue or red based on whether they were positively or negatively enriched in shAR versus shCtrl with an FDR < 0.05.

To understand the relationship between Stat5 and AR more broadly, we used RNA sequencing (RNA-seq) in CWR22Rv1 cells to compare transcriptome-wide effects of Stat5 inhibition with IST5-002 and AR inhibition with lentiviral shRNA (shAR) (Fig. 6A and fig. S5, A to C). IST5-002 treatment regulated a broader repertoire of genes in CWR22Rv1 cells than AR knockdown (Fig. 6B and data files S1 to S4). Focusing on a set of 17 defined AR target genes, Stat5 inhibition with IST5-002 reduced AR activity to a similar degree as AR knockdown with shRNA (Fig. 6, C and D). Conversely, only IST5-002 reduced the expression of defined Stat5 target genes *CCND1*, *SOCS2*, *PIM1*, *RHOH*, and *CISH* (Fig. 6C) (*53*–*55*).

Using gene set enrichment analysis (GSEA) (*55*), we analyzed the enrichment of all 50 hallmark gene sets in the mSigDB molecular signatures database (*56*) (data files S5 to S8). Notably, of the 19 hallmark gene sets that were positively enriched in CWR22Rv1 cells expressing AR versus control shRNA (blue in Fig. 6E, top), 16 (84%) were similarly positively enriched in CWR22Rv1 cells treated with IST5-002 versus vehicle control (blue in Fig. 6E, bottom). An example of one of these gene sets was HALLMARK_APOPTOSIS, which is a set of 161 genes that mediate programmed cell death. Furthermore, of the 18 hallmark gene sets that were negatively enriched in CWR22Rv1 cells expressing AR versus control shRNA, 12 (67%) were similarly negatively enriched in CWR22Rv1 cells treated with IST5-002 versus vehicle control (red in Fig. 6C). An example of one of these gene sets was HALLMARK_E2F_TARGETS, which is a set of 200 cell cycle–related target genes of E2F transcription factors. We also performed the reciprocal analysis and found that 16 of 23 (69%) of gene sets positively enriched in CWR22Rv1 cells treated with IST5-002 versus vehicle control were similarly positively enriched in CWR22Rv1 cells expressing AR versus control shRNA (blue in fig. S5D), and 12 of 14 (86%) of gene sets negatively enriched in CWR22Rv1 cells treated with IST5-002 versus vehicle control were similarly negatively enriched in CWR22Rv1 cells expressing AR versus control shRNA (red in fig. S5D).

Last, we used GSEA to analyze the enrichment of all 189 oncogenic signature gene sets in mSigDB (data files S5 to S8) and confirmed that the majority of these gene sets displayed similar positive or negative directionality of regulation when comparing AR inhibition to Stat5 inhibition (fig. S5, E and F). Collectively, these results demonstrate that Stat5 regulates AR expression and activity in PC cells, which manifests in Stat5 and AR having similar positive and negative regulatory effects on gene sets that reflect fundamental biological and oncogenic processes.

To explore the mechanism by which Stat5 regulates *AR* gene transcription, we pursued a hypothesis that Stat5 binds *AR* gene regulatory elements. To test this, we performed Stat5 chromatin immunoprecipitation (ChIP) and DNA sequencing (ChIP-seq) in 22Rv1 cells. Prl treatment stimulated Stat5 binding to the transcription start sites of Stat5 target genes *CISH, SOCS2, BCL6*, and *GPCPD1* (fig. S6, A to D). However, there was no evidence for Stat5 binding to the *AR* transcription start-site, or to regions extending 800 kb upstream and downstream of the AR gene body, which includes a known enhancer located 650 kb upstream of *AR* (fig. S6E) (*14*, *15*, *57*). These results indicate that Stat induction of *AR* gene transcription does not occur through a simple mechanism of Stat5 binding to *AR* gene regulatory elements.

### Stat5 inhibitor IST5-002 decreases the fraction of viable prostate cancer cells through suppression of the Stat5-AR axis

**Fig. 7.**
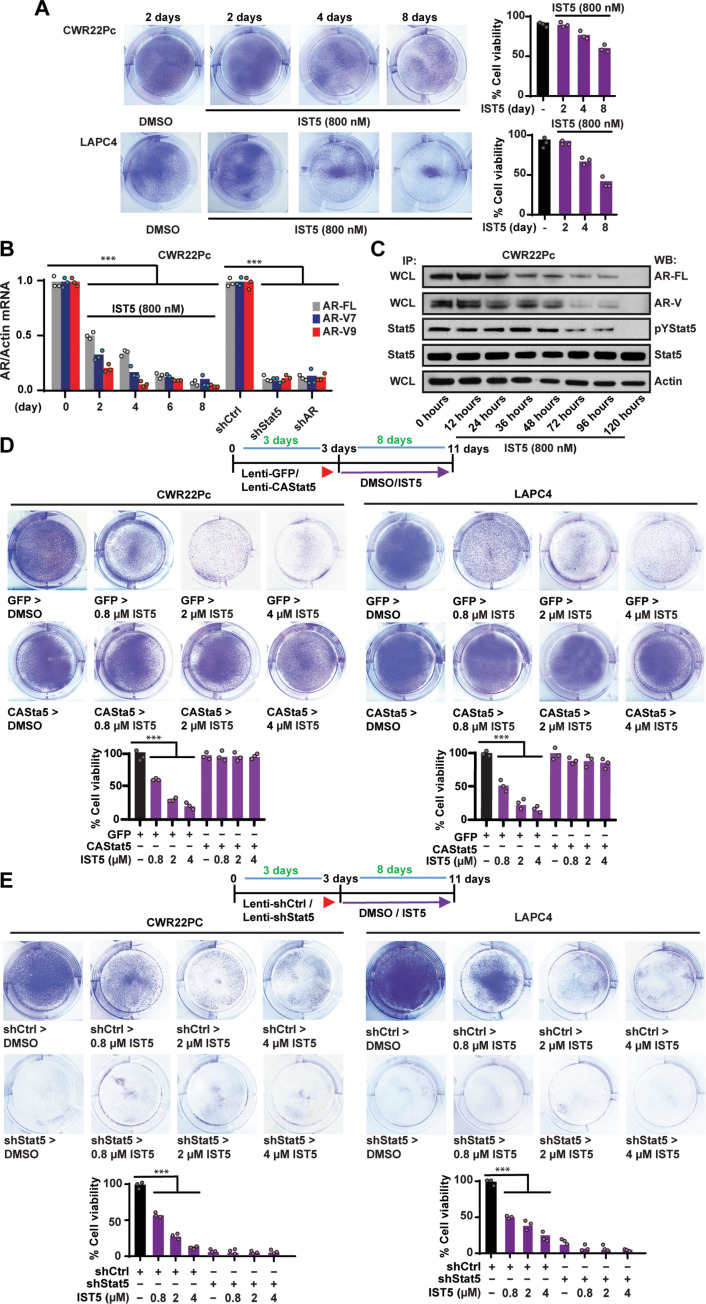
Stat5 inhibitor IST5-002 suppresses PC cell viability by down-regulation of Stat5 levels. (**A**) CWR22Pc cells were treated with 800 nM IST5-002 (IST5) or DMSO up to 8 days, as indicated, and the fraction of live cells were determined by crystal violet staining of the attached surviving cells at each time-point and counted. (**B**) AR-FL, AR-V7, and AR-V9 mRNA levels were determined by qRT-PCR in cells from parallel wells at indicated time points with cells transduced with lentivirus expressing shAR, shStat5, or shCtrl for 72 hours as comparison. (**C**) AR protein levels evaluated by WB of WCLs with actin as a loading control. The levels of Stat5 activation were determined by the IP of Stat5 followed by WB for pStat5a/b and total Stat5. (**D**) CAStat5 or GFP was lentivirally expressed in CWR22Pc and LAPC4 cells for 3 days followed by treatment of the cells with IST5-002 for 8 days at indicated concentrations. The fractions of live attached cells in each treatment group were determined by crystal violet staining and counting. (**E**) Stat5 was suppressed by lentiviral expression of Stat5 shRNA or shCtrl for 3 days before treatment of the cells with increasing concentrations of IST5-002 followed by the determination of the fractions of viable cells in each treatment group. Hypothesis tests were performed using ANOVA with post hoc testing using the Student’s *t* test with Bonferroni multiplicity corrections. Significance levels are indicated for relevant hypotheses as *P < 0.05, **P < 0.01, and ***P < 0.001.

AR is the predominant molecular target for pharmacological non-chemotherapeutic PC therapies. Having established that Stat5 inhibition by IST5-002 suppresses AR expression and activity at sub-micromolar concentrations in PC cells, we next assessed the capability of IST5-002 to suppress PC cell viability by decreasing the AR. We have previously shown that genetic or pharmacological Stat5 inhibition by IST5-002 induces apoptotic death of PC cells (*31*, *32*, *36*). Treatment of PC cells with 800 nM IST5-002 reduced the fraction of alive attached PC cells at 8 days (Fig. 7A). This was accompanied by reduced levels of AR-FL and AR-V mRNA and protein levels as determined by qRT-PCR and immunoblotting (Fig. 7, B and C). To investigate whether this reduction in the number of live cells was mediated by suppression of Stat5, CAStat5 was expressed in CWR22Pc and LAPC4 cells followed by treatment with IST5-002. CAStat5 counteracted the ability of IST5-002 to reduce the number of live cells, even at concentrations as high as 4 μM IST5-002 (Fig. 7D). To identify potential off-target effects of IST5-002, Stat5 was depleted by lenti-shStat5 followed by IST5-002 treatment. As shown in Fig. 7E, IST5-002 was not able to further decrease the numbers of live CWR22Pc cells after Stat5 knockdown, which indicated that IST5-002 acts through Stat5 to suppress PC growth.

**Fig. 8.**
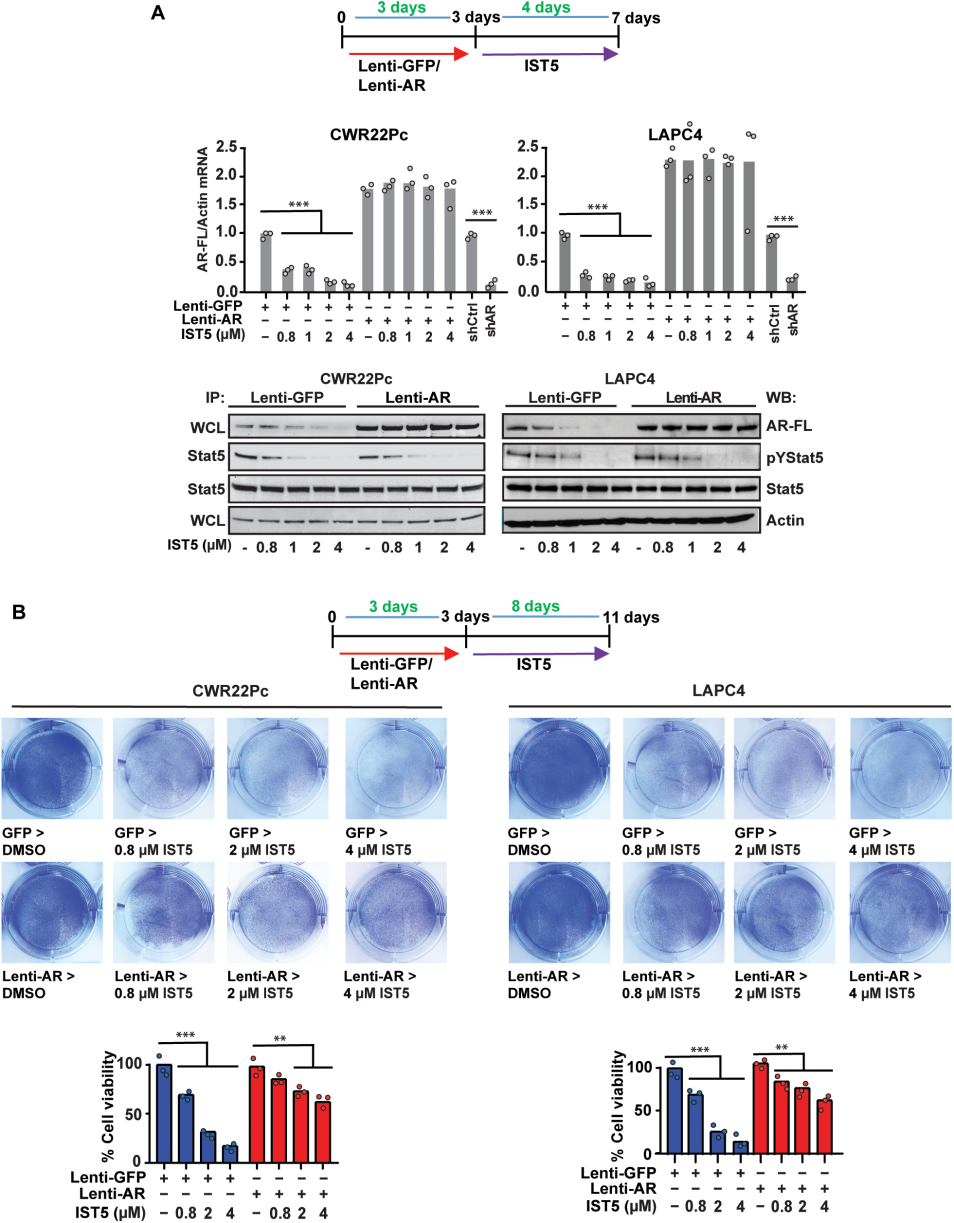
Stat5 inhibitor IST5-002 suppresses the viability of PC cells by inhibition of Stat5-AR levels. (**A**) AR-FL or GFP was lentivirally expressed in CWR22Pc and LAPC4 cells for 3 days before treatment of the cells with increasing concentrations of IST5-002 for 4 or (**B**) 8 days. AR-FL mRNA and protein levels were determined by qRT-PCR and WB, respectively. Activation levels of Stat5 were evaluated by the IP of Stat5 followed by WB for pStat5 and total Stat5. (B) The fractions of live attached cells in each treatment group were determined by crystal violet staining and counting. Hypothesis tests were performed using ANOVA with post hoc testing using the Student’s *t* test with Bonferroni multiplicity corrections. Significance levels are indicated for relevant hypotheses as *P < 0.05, **P < 0.01, and ***P < 0.001.

Because pharmacological Stat5 inhibition suppresses PC cell growth, we next sought to evaluate whether IST5-002 reduction of PC cell viability was caused by suppression of AR levels. In these experiments, AR was overexpressed by lentivirus in CWR22Pc and LAPC4 cells with lenti-GFP as control, followed by IST5-002 treatment (Fig. 8, A and B). IST5-002 did not suppress exogenously expressed AR mRNA and protein levels in CWR22Pc and LAPC4 PC cells (Fig. 8A) suggesting the involvement of endogenous regulatory regions affecting the *AR* gene transcription being critical for induction by Stat5. At the same time, overexpression of the AR before IST5-002 treatment of the CWR22Pc and LAPC4 cells was able to rescue approximately 40 to 50% of CWR22Pc and LAPC4 cells from IST5-002–induced cell death (Fig. 8B). These data support the concept that IST5-002 suppresses PC cell viability via inhibition of the Stat5-AR axis, but IST5-002 suppression of PC cell viability is not entirely mediated by AR inhibition.

### IST5-002 suppresses AR levels and PC growth in both non-castrate and castrate settings in vitro and in vivo in PC tumors

**Fig. 9.**
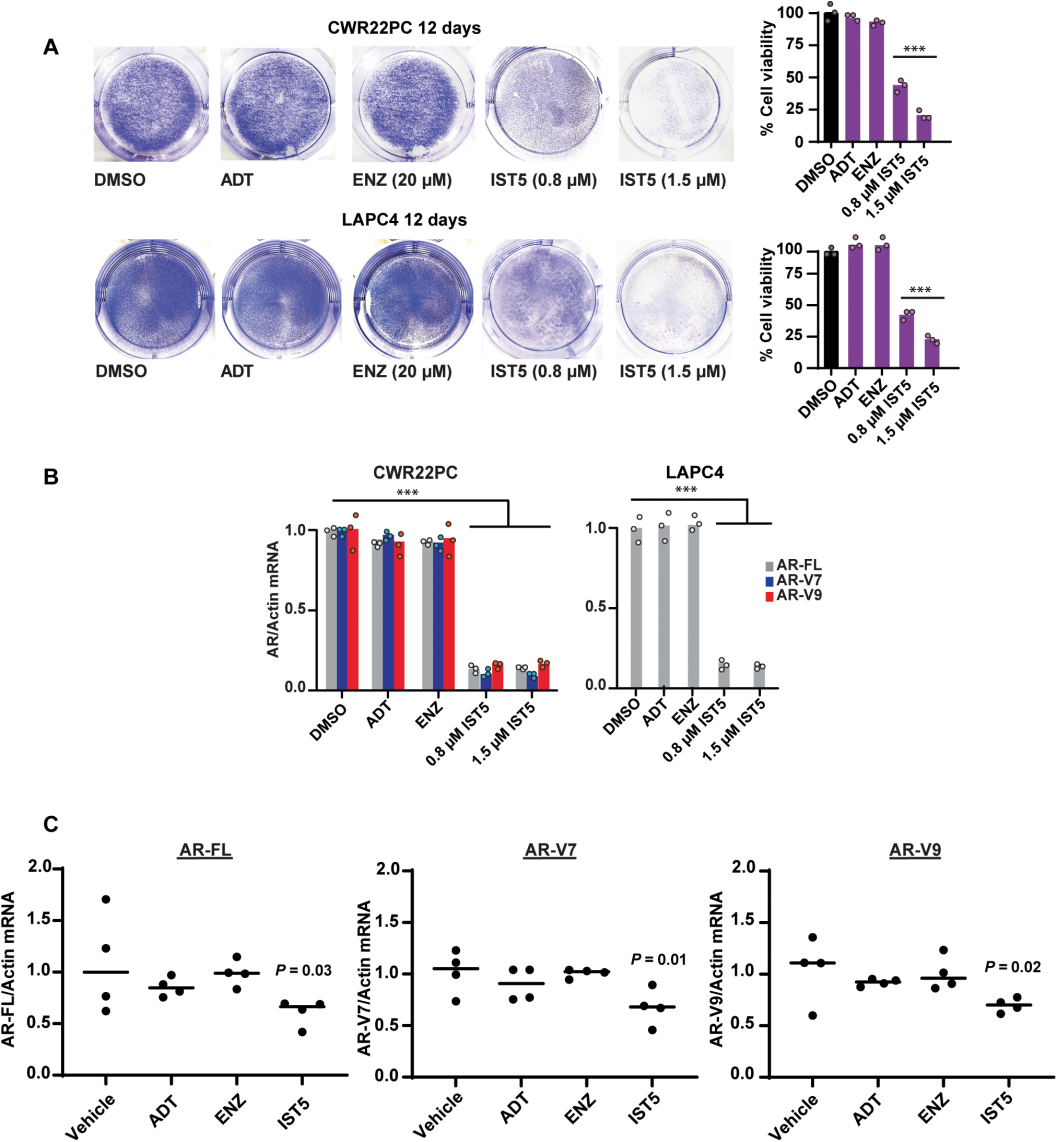
IST5-002 (IST5) suppresses AR levels and growth in a non-castrate setting in PC in vitro and in vivo. (**A**) CWR22Pc and LAPC4 cells were treated with vehicle (DMSO), ADT (withdrawal of androgens), ENZ (20 μM), or IST5-002 (IST5) (800 and 1500 nM) for 12 days followed by the determination of the fractions of live attached PC cells by crystal violet staining and counting and (**B**) evaluation of AR-FL, AR-V7, and AR-V9 mRNA levels by qRT-PCR. (**C**) CWR22Pc cells were inoculated subcutaneously into flanks of castrated athymic nude mice supplied with sustained-release DHT pellets. Mice were surgically castrated or treated daily with vehicle, ENZ (30 mg/kg), or IST5-002 (50 mg/kg) for 32 days. AR-FL, AR-V7, and AR-V9 mRNA levels were evaluated by qRT-PCR from the endpoint tumors. Hypothesis tests were performed using ANOVA with post hoc testing using the Student’s *t* test with Bonferroni multiplicity corrections. Significance levels are indicated for relevant hypotheses as *P < 0.05, **P < 0.01, and ***P < 0.001.

Having established that inhibition of active Stat5 signaling suppresses PC cell viability through decreasing AR, we compared the in vitro efficacy of ADT versus IST5-002 in decreasing the fraction of viable PC cells. ADT was conducted by the withdrawal of androgens [charcoal-stripped fetal bovine serum (cs-FBS), no dihydrotestosterone (DHT)] or by administration of the anti-androgen ENZ to CWR22Pc and LAPC4 cells and compared to IST5-002 treatment of cells with vehicle as a control. IST5-002, even at 800 nM, was more potent than ADT or high micromolar concentrations of ENZ in reducing alive CWR22Pc and LAPC4 cell numbers (Fig. 9A). In addition to PC cell viability, IST5-002 reduced mRNA levels of the AR-FL or AR-Vs robustly when compared to androgen withdrawal (ADT) or ENZ (Fig. 9B). We have previously shown that IST5-002 is highly efficacious in inhibiting androgen-sensitive CWR22Pc xenograft tumor growth in vivo when compared to ENZ or ADT (fig. S7A) (*36*, *40*). In these experiments, CWR22Pc cells were subcutaneously inoculated to castrated athymic nude mice supplied with sustained-release DHT pellets to normalize circulating androgen levels. The mice were treated with ENZ or IST5-002 daily for 32 days with vehicle or surgical castration as control groups (Fig. 9C and fig. S7A). In endpoint tumors from these androgen-sensitive CWR22Pc xenograft growth experiments, IST5-002 suppressed FL-AR and AR-V mRNA levels when compared to ENZ or ADT (Fig. 9C), which corresponded with reduced PC xenograft tumor growth in vivo (fig. S7A). Together, these data demonstrate that IST5-002 inhibits AR mRNA levels and CWR22Pc tumor growth in a non-castrate setting in vivo.

**Fig. 10.**
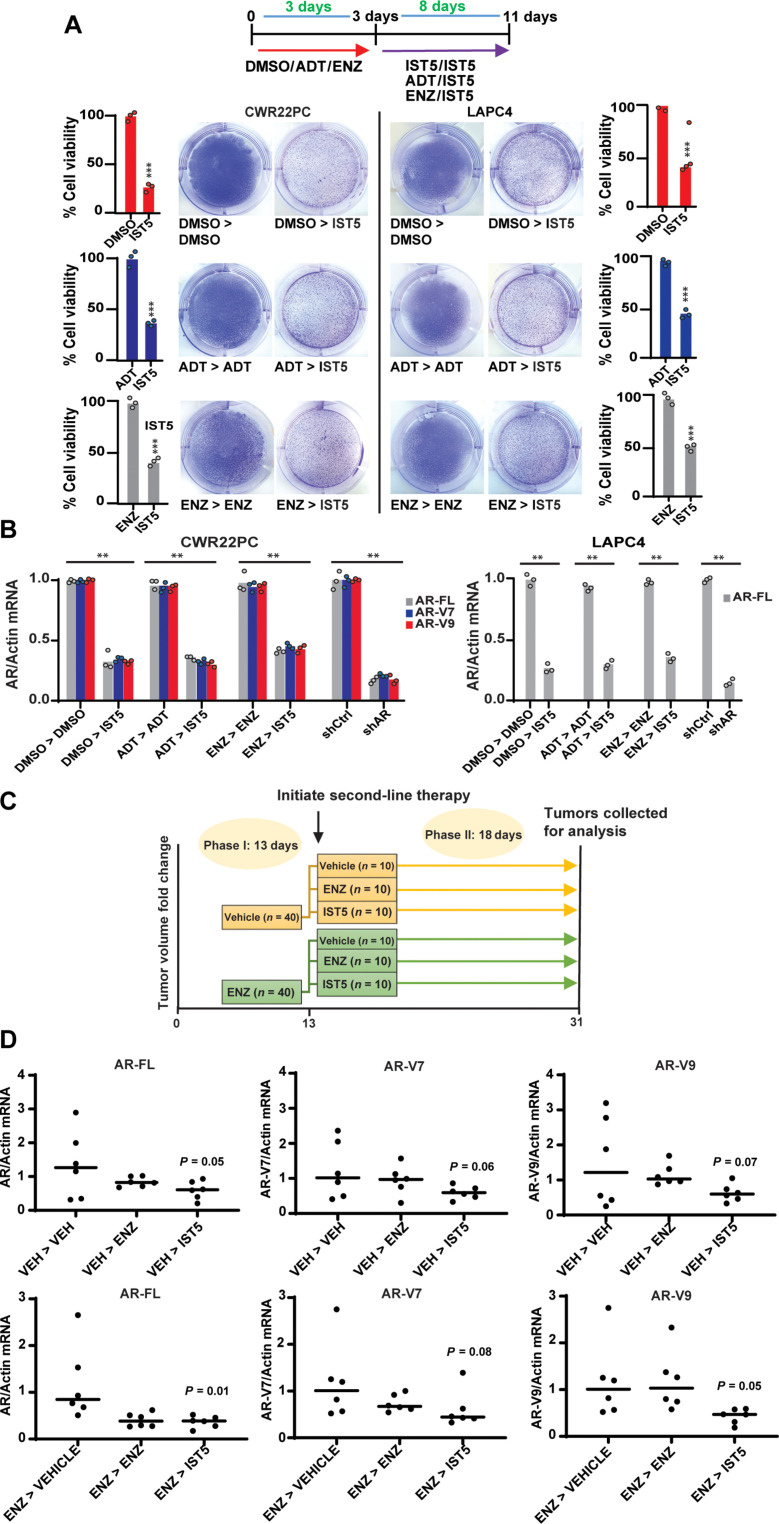
IST5-002 (IST5) suppresses AR levels and growth in a castrate setting in PC in vitro and in vivo. (**A**) CWR22Pc and LAPC4 cells were treated with vehicle (DMSO), ADT (withdrawal of androgens), or ENZ (20 μM) for 3 days followed by continuation with DMSO, ADT, ENZ (20 μM), or IST5-002 (800 nM) for 8 days. The fractions of surviving attached cells were determined by crystal violet staining and counting. (**B**) AR-FL, AR-V7, and AR-V9 mRNA levels of cells in parallel wells were evaluated by qRT-PCR. (**C**) Experimental design for the sequential therapy of the xenograft tumors in vivo. A two-phase in vivo experiment using vehicle or ENZ as first-line therapy (phase 1, 13 days) and vehicle, ENZ, or IST5-002 as second-line therapy (phase 2, 18 days). On day 31, mice were euthanized and CWR22Pc xenograft tumors were collected for analyses. (**D**) AR-FL, AR-V7, and AR-V9 mRNA levels were determined in the endpoint tumors by qRT-PCR. Hypothesis tests were performed using ANOVA with post hoc testing using the Student’s *t* test with Bonferroni multiplicity corrections. Significance levels are indicated for relevant hypotheses as *P < 0.05, **P < 0.01, and ***P < 0.001.

To assess therapeutic efficacy of sequential targeting of AR and Stat5 in PC cells in vitro, CWR22Pc and LAPC4 cells were first treated with vehicle [dimethyl sulfoxide (DMSO)], ADT, or ENZ for 3 days followed by continued treatment with either DMSO, ADT, or ENZ versus IST5-002 (800 nM) for 8 days (Fig. 10A). The assessment of viable cells and AR mRNA levels at the end of this treatment regimen showed that IST5-002 decreased the number of live PC cells and mRNA levels of AR-FL and AR-Vs following pharmacological targeting of the AR in vitro (Fig. 10, A and B) To examine the efficacy of therapeutic targeting of Stat5 in CRPC after ENZ treatment in vivo, we used CWR22Pc tumors known to initially display androgen-sensitive growth and regress upon castration, but later recur as castrate-resistant tumors (*58*). We have previously shown that IST5-002 has high efficacy in reducing CWR22Pc xenograft tumor growth after the development of ENZ resistance in vivo (*40*). In these experiments (*40*), CWR22Pc cells were inoculated subcutaneously in the flanks of athymic nude mice followed by first-line therapy of vehicle or ENZ daily until the emergence of resistance to ENZ occurred (day 13), followed by randomization and switch to a second-line therapy (vehicle, ENZ or IST5-002) daily for additional 18 days, as depicted in Fig. 10C. In endpoint ENZ-resistant and ENZ-naïve tumors from these experiments, AR-FL and AR-V mRNA levels were decreased by IST5-002 (Fig. 10, C and D). Furthermore, Stat5 inhibition by single-agent treatment with IST5-002 suppressed androgen-sensitive PC xenograft tumor growth with efficacy comparable to ENZ (fig. S7B). IST5-002 treatment of CWR22Pc tumors that had developed ENZ resistance resulted in a robust suppression of CRPC tumor growth (fig. S7C). In summary, the inhibition of Stat5 by IST5-002 reduced AR-FL and AR-V mRNA levels in both androgen-sensitive and castrate-resistant CWR22Pc tumors, which was accompanied by decreased CRPC tumor growth.

## DISCUSSION

AR is critical for the initial development of PCs and drives the growth of established PCs. This dependency is exploited by ADT, which, however, is compromised by acquired genetic changes occurring in the AR that permit continued activation in the absence of the ligand. The present study uncovers an unexpected role of active Stat5 signaling, a known promoter of PC growth and clinical progression (*31*–*46*), as a potent inducer of *AR* gene transcription in PC cells. This finding opens new therapeutic avenues to directly target AR levels in PC via Stat5 inhibition with the potential to achieve a prolonged therapeutic response. Pharmacological inhibition of Stat5 activity in PC cancer by a specific small-molecule Stat5 inhibitor, IST5-002 (*36*, *39*, *40*, *47*), suppressed Stat5-regulated genes, and *AR* and AR-regulated genes resulting in reduced PC cell viability and tumor growth.

Genetic knockdown of Stat5 protein robustly and consistently suppressed both AR-FL and AR splice variant mRNA levels in human PC cell lines regardless of the status of somatic *AR* mutations or *AR* locus amplification. The extent of AR and PSA suppression by Stat5 depletion in PC was largely comparable to levels achieved by direct genetic knockdown of AR itself. In addition, Stat5 knockdown suppressed AR mRNA levels in PC lines (*23*, *24*) genetically engineered to express AR gene rearrangements under endogenous *AR* locus previously shown to occur in clinical PCs (*24*, *49*). The decrease of AR-FL and AR-V mRNAs in PC was reflected at reduced AR protein levels and suppression of the AR target gene PSA indicating that Stat5 depletion leads to suppression of AR signaling in PC cells. Activated Stat5 induced AR mRNA and protein levels in PC cells. This was demonstrated by CA Stat5 overexpression inducing mRNA and protein levels of AR and PSA. At the same time, cytokine activation of the endogenous Jak2-Stat5 signaling pathway in PC cells induced AR-FL and AR-V mRNA levels, which indicated that ligand-induced activation of naturally occurring Stat5 is capable of inducing AR mRNA and protein expression in PC. We have previously shown that the knockdown of Stat5 in PC cells decreases the protein stability of the AR (*48*). The data presented here show that while Stat5 depletion induces proteasomal degradation of AR, activation of Stat5 does not increase AR protein stability. Stat5 depletion did not affect AR mRNA and protein levels in AR-positive breast cancer cells, which suggests that Stat5 induction may be tissue-specific. This finding is intriguing and warrants future studies evaluating the concept in various AR-positive normal and malignant cell types such as liver, urogenital, cardiovascular, and neuronal tissues which are affected by the traditional inhibitors of AR activity. These future studies will also need to investigate the mechanisms causing tissue specificity which may include the epigenetic modification of *AR* regulatory regions or PC-specific Stat5-regulated AR repressors. Peptide hormones and growth factors that induce Stat5 activation in PC tissue include locally expressed Prl acting in an autocrine/paracrine manner (*50*, *52*, *59*–*62*), growth hormone, epidermal growth factor, and granulocyte-macrophage colony-stimulating factor (*63*–*74*). Collectively, these findings demonstrate that the presence of Stat5 protein in PC cells improves AR protein stability, but the activation of this signaling pathway induces AR-FL and AR splice variant mRNA and protein levels, a finding that can be exploited for therapeutic purposes.

The mechanisms underlying Stat5 induction of the mRNA levels of AR-FL and AR-Vs may involve either direct up-regulation of *AR* gene transcription or Stat5 suppression of an AR repressor in PC cells. The fact that ongoing protein synthesis was not required for CAStat5 to induce AR mRNA levels suggests a mechanism not mediated by the synthesis of other proteins. In addition, CAStat5 did not slow the kinetics of AR mRNA decay ruling out Stat5 induction of AR mRNA stability in PC cells. Lentiviral Stat5 knockdown robustly down-regulated and CAStat5 up-regulated the levels of newly synthetized nascent AR pre-mRNA and nascent spliced AR-FL and AR-V mRNAs. At the same time, as expected, lentiviral shAR knockdown did not suppress nascent AR pre-mRNA levels supporting the finding that active Stat5 induces the transcription of the AR gene in PC cells. However, ChIP-seq analysis with an anti-Stat5 antibody did not reveal evident Stat5 binding sites in the *AR* gene, or surrounding regulatory regions. The region that was analyzed for Stat5 binding included a transcriptional enhancer located ~650 kb upstream of the AR gene body, which is important for the transcription of the *AR* gene in CRPC cells (*14*, *57*, *75*). While these data do not rule out a direct effect of Stat5 on *AR* gene transcription, they do indicate that this would occur over a large genomic distance. Future studies are warranted to characterize Stat5 binding sites that interact with the AR transcription start site over large genomic distances. In addition, these future studies should also consider the mechanism(s) by which Stat5 activation indirectly relieves *AR* transcriptional repression.

Studies of PC lineage plasticity found that up-regulation of Jak/Stat signaling is an early event that promotes mixed basal/luminal and/or stem cell–like identity of PC cells characterized by reduced AR expression and AR pathway dependence (*76*–*78*). Accordingly, the dual Jak1/2 inhibitor ruxolitinib applied at a high dose or Jak1 knockout restored AR expression and activity in various models of *AR*-null PC (*76*, *77*). Noteworthy, these effects of Jak/Stat signaling were only observed in a background of *TP53* and *RB1* deficiency. Furthermore, loss-of-function screening of individual Stat family members showed that ENZ-resistant growth of these *TP53/RB1*-null models was supported specifically by Stat1 (*77*). Our work identifying active Stat5 signaling as a mechanism that reinforces AR expression in PC cells suggests divergent and context-specific effects of individual Stat family members on AR expression and AR pathway dependence in PC. Additional studies are warranted to dissect the roles of individual Stat family members on the maintenance versus erosion of AR expression in PC cells with varying genomic backgrounds.

One of the key results presented in the work shows that a specific small-molecule Stat5 inhibitor IST5-002, which inhibits both phosphorylation and dimerization of Stat5 (*36*, *39*), suppressed AR mRNA and protein levels in preclinical PC models and patient-derived PC samples with high potency. IST5-002 suppressed AR-FL and AR-V mRNA and protein levels in sub-micromolar concentrations in human PC cell lines. Down-regulation of the levels of nascent AR-FL and AR-V pre-mRNAs indicates the suppression of the *AR* gene transcription by IST5-002 in PC. At the same time, specificity studies conducted by simultaneous overexpression of CAStat5 or Stat5 depletion by shStat5 with IST5-002 treatment of PC cells demonstrated that IST5-002 regulation of AR mRNA levels in PC cells is mediated through Stat5. Extending to human tissues, IST5-002 down-regulated AR-FL and AR-V mRNA levels in six patient-derived clinical PCs cultured ex vivo in tumor explant cultures suggesting potential efficacy in clinical PC.

A functional outcome of IST5-002 down-regulating AR-FL and AR-V mRNA and protein levels was that IST5-002 broadly reduced AR pathway activity in PC cells. This was evident from RNA-seq profiling of transcriptome-wide gene expression changes in PC cells treated with IST5-002, and comparing this to genome-wide gene expression changes caused by genetic knockdown of AR by lentiviral shAR. Additional genome-wide similarities were noted in the effects of IST5-002 versus AR knockdown on the regulation of multiple hallmarks and oncogenic gene sets in the mSigDB molecular signatures database. This, coupled with the findings that Stat5 target genes were responsive to IST5-002 but not AR knockdown and that a larger repertoire of genes was responsive to IST5-002 compared with AR knockdown, strongly supports a conclusion that Stat5 is an upstream regulator of AR mRNA levels in PC cells.

IST5-002 suppressed the fraction of live attached PC cells at sub-micromolar concentrations in parallel with suppression of AR-FL and AR-v mRNA and protein levels, which was counteracted by overexpression of activated Stat5 (CAStat5). We and others have previously shown that both genetic and pharmacological Stat5 inhibition elicits extensive apoptotic death of not only AR-positive PC cells (*32*, *37*, *38*) but also AR-negative DU145 cells (*33*). Here, the simultaneous introduction of AR to PC cells with IST5-002 treatment was able to partially rescue AR-positive PC cells CWR22Pc and LAPC4. Together, these findings suggest that Stat5 inhibition by IST5-002 affects additional pathways critical for PC cell growth and survival capabilities, which is a concept also supported by the RNA-seq analysis showing a broader set of genes regulated by IST5-002 compared to AR knockdown. Last, IST5-002 repressed AR mRNA levels and the fraction of viable PC cells also in a castrate-resistant setting when applied after ADT or ENZ in vitro and in CWR22Pc xenograft tumors in vivo.

Overall, the identification of the active Stat5 signaling pathway as an inducer of AR-FL and its various spliced and mutated forms has high translational significance in opening a new therapeutic modality for PC. In addition, the concept may have future implications on PDX modeling of human PC in mice given the ligand/host incompatibilities in Prl/Prl-receptor/Stat5 activation (*79*). Lead optimization and design of oral formulations of the IST5-002 will enable its entrance to the clinical development pipeline.

## MATERIALS AND METHODS

### Cell lines and reagents

LNCaP, CWR22Rv1 [from American Type Culture Collection (ATCC)], CWR22Pc (*58*), R1-AD1 (*49*), R1-D567 (*49*), R1-I567 (*49*), and R1-X-11 (*49*) cells were cultured in RPMI 1640 growth media (Mediatech) containing 10% FBS (Gemini) and penicillin/streptomycin (50 IU/ml and 50 μg/ml, respectively; Mediatech) following the procedures we have previously described (*32*, *37*, *40*, *47*, *48*). VCaP and LAPC4 (from ATCC) were cultured under the same conditions, with the substitution of RPMI 1640 for Dulbecco’s modified Eagle’s medium and Iscove’s modified Dulbecco’s medium (Mediatech), respectively. VCaP, LNCaP, LAPC-4, and CWR22Pc cells were cultured in the presence of DHT (Sigma-Aldrich; LNCap: 0.5 nM, LAPC-4: 1 nM, VCaP and CWR22Pc: 0.8 nM). CWR22Pc subline–expressing AR-F876L was cultured in RPMI 1640 supplemented with ENZ (10 μM). All cell lines were regularly authenticated by observation of cell morphology, androgen responsiveness, and expression of cell line–specific markers and tested for *Mycoplasma* contamination (PCR Mycoplasma Detection Set; Takara Bio Inc.) every 3 months. ENZ was purchased from MedChem Express, CHX and MG132 from Calbiochem, actinomycin D from Sigma-Aldrich, and IST5-002 (*36*, *39*) was provided by Fox Chase Chemical Diversity Center (Doylestown, PA). Recombinant human Prl was obtained from the NIDDK Hormone and Peptide Program (Torrance, CA).

### Lentiviral production and transduction of cells

The RNAi Consortium (TRC) pLKO.1 lentiviral vector containing shRNA targeting AR, Stat5a, Stat5b, Stat3, or scrambled control sequences was purchased from Open Biosystems, Dharmacon. For RNA-seq experiments, lentivirus expressing control shRNA and two independent shRNAs targeting AR was purchased from Horizon Discovery (shC = RHS4348, shAR-1 = V2LHS_239574, shAR-2 = V2LHS_149847). CAStat5a (S710F), CAStat5b (S715F) (*35*), and GFP sequences were cloned into pLCP plasmid using InFusion Cloning kit under CMV promoter and AR-FL under SV40 promoter (*20*). Second-generation VSV-G pseudo-typed high-titer lentiviruses were generated by transient cotransfection of HEK293 cells, with a three-plasmid combination as follows: lentiviral vector (9-μg pLKO.1) containing shRNA or cDNA of interest, 10-μg pHR’8.2ΔR packaging plasmid and 1-μg pCMV-VSV-G envelope plasmid using Lipofectamine 2000 (Life Technologies) in Opti-MEM (Life Technologies). In all experiments, lentivirus-containing culture media were changed to regular growth media 16 hours after the initiation of lentiviral gene transduction. This time point served as the start for counting the length of the lentiviral gene expression illustrated in fig. S1A. PC cells were transduced with 200 to 250 μl of lentiviral shRNA supernatant in the presence of polybrene (1:1000; Sigma-Aldrich) to induce >80% knockdown or activation of the protein of interest.

### Protein solubilization, immunoprecipitation, and immunoblotting

Cell pellets were solubilized in lysis buffer [10 mM tris-HCl (pH 7.6), 5 mM EDTA, 50 mM sodium chloride, 30 mM sodium pyrophosphate, 50 mM sodium fluoride, 1 mM sodium orthovanadate, 1% Triton X-100, 1 mM phenylmethylsulfonyl fluoride, aprotinin (5 μg/ml), pepstatin A (1 μg/ml), and leupeptin (2 μg/ml)]. Protein concentrations of clarified cell lysates were determined by the simplified Bradford method (Bio-Rad) followed by immunoprecipitation with Stat5a/b antibodies at 4°C for 1 hour, followed by incubation of the samples with Sepharose A beads for 2 hours at 4°C with end-to-end rotation. The samples were centrifuged at 1000 rpm at 4°C for 1 min and the supernatants were discarded. The samples were separated by SDS–polyacrylamide gel electrophoresis and transferred to a polyvinylidene difluoride membrane followed by immunoblotting with specific antibodies (table S1). The immunoreaction was detected by horseradish peroxidase-conjugated secondary antibodies followed by enhanced chemiluminescence (GE Healthcare).

### mRNA stability assay

CWR22Rv1 cells were seeded in RPMI 1640 containing 10% FBS for 24 hours followed by transduction with lentivirus encoding GFP or CAStat5a/b for 72 hours followed by treatment with 5 μM actinomycin D (Sigma-Aldrich), as indicated. Cells were harvested in guanidinium thiocyanate buffer at the indicated time points and RNA was extracted and analyzed by qRT-PCR for AR-FL.

### Nascent RNA labeling and isolation

Nascent transcripts were labeled with biotin and subjected to streptavidin pull-down using the Click-iT Nascent RNA Capture Kit (Life Technologies) according to the manufacturer’s specifications. Briefly, CWR22Rv1 cells were seeded in RPMI 1640 containing 10% FBS for 24 hours followed by transduction with lentivirus encoding shCtrl, shStat5, shAR, GFP, or CAStat5a/b. In parallel treatment groups, CWR22Rv1 cells were treated with actinomycin D (5 μM), DMSO, or IST5-002 (*36*, *39*) at indicated concentrations for 24, 48, or 72 hours. Cells were pulsed with 5-ethynyl uridine (5EU) for an additional hour to label nascent transcripts, and then harvested in TRIzol (Life Technologies). Total RNA was then subjected to a Click-iT chemistry reaction which attached a biotin molecule to 5EU-labeled nascent transcripts. RNA was reprecipitated, and then bound to streptavidin-conjugated magnetic beads and washed 10 times to remove unlabeled transcripts, leaving only biotin-5EU–labeled nascent RNA attached to the beads. First-strand cDNA synthesis was performed directly on RNA:bead conjugates using the SuperScript VILO cDNA synthesis kit (Life Technologies) according to the manufacturer’s specifications, then subjected to qRT-PCR.

### Quantitative real-time RT-PCR

Total RNA was reverse-transcribed using the SuperScript III First-Strand Synthesis System (Invitrogen). Quantitative PCR was carried out using AR-FL (EXON3 F: 5′- AAC AGA AGT ACC TGT GCG CC-3′ and EXON4 R: 5′-TTC AGA TTA CCA AGT TTC TTC AGC -3′), AR-V7 (EXON3 F: 5′-AAC AGA AGT ACC TGT GCG CC-3′ and CE3 R: 5′-TCA GGG TCT GGT CAT TTT GA-3′), AR-V9 primers (EXON3 F: 5′-AAC AGA AGT ACC TGT GCG CC-3′ and CE5 R: 5′-GCA AAT GTC TCC AAA AAG CAG C-3′), Stat5 (F: 5′- ACT GCT AAA GCT GTT GAT GGA TAC and R: 5′- TGA GTC AGG GTT CTG TGG GTA), PSA primers (F: 5′- GGG ACA ACT TGC AAA CCT GC and R: 5′- GTA TCT GTG TGT CTT CTG AGC), and actin (F: 5′- CAG CCA TGT ACG TTGCTA TC and R: 5′- CTT CAT GAG GTA GTC AGT CA) HotStart-IT SYBR Green One-Step qRT-PCR Master Mix (Affymetrix). Relative changes in expression levels were determined by a comparative CT method using the formula 2-ΔΔCT; where CT is the threshold cycle of amplification and expressed per the levels of actin mRNA.

### RNA-seq of Stat5-regulated genes in PC cells

CWR22Rv1 cells were transduced with lentivirus encoding shCtrl, shAR-1, or shAR-2, followed by two sequential rounds of selection with puromycin dihydrochloride (2 μg/ml; Gibco, A11138-03). Infected cells were expanded and seeded in six-well plates at 500,000 cells per well and grown for 72 hours in normal RPMI 1640 + 10% FBS media. The cells infected with shCtrl lentivirus were further expanded, seeded in six-well plates, and treated 24 hours after seeding with DMSO (vehicle control) or 0.8 μM IST5-002. Media were refreshed every 24 hours for a total treatment time of 72 hours. Total RNA was extracted from cells using the ReliaPrep RNA Miniprep System (Promega). RNA was submitted to University of Minnesota Genomics Center (UMGC) for RNA-seq library synthesis using a TruSeq Stranded mRNA kit (Illumina) according to the manufacturer’s instructions. RNA-seq libraries were subjected to 2 × 150 base pair (bp) paired-end sequencing on an Illumina NovaSeq 6000 system.

### Identification of differentially expressed genes in RNA-seq data

Fastq files containing 150-bp paired-end reads were aligned to the hg19 reference genome using HiSat2 (v. 2.1.0) (*80*). Subread (v. 2.0.3) (*81*) was used to quantify gene expression using version 100 GRCh38 annotation from Ensembl (*82*). Count data were filtered to only keep genes that had a counts per million (cpm) value greater than 1 cpm in at least two samples across all experimental conditions. Differential expression was calculated using the glmQLFTest function in edgeR (v. 3.36.0) (*83*, *84*), The Benjamini-Hochberg method was used for multiple hypothesis testing correction. An adjusted *P* value = 0.05 was used as a differential expression significance threshold (*80*–*85*).

### Gene set enrichment analysis

Ranked gene lists for GSEA were generated using the following two-step transformation. First, unadjusted *P* values from the differential expression tests were transformed by −log_10_, then the −log_10_ *P* values for each gene were multiplied by either +1 or −1 depending on the sign of the expression fold change for that gene (positively regulated genes are multiplied by +1 and negatively regulated genes are multiplied by −1). This ranked gene list was used for “Pre-ranked” GSEA analysis using the GSEA java program (v. 4.2.3) (*85*). For significance testing, 10,000 permutations were tested instead of the default of 1000 to better control for false discoveries. To enable reproducible testing results, a seed of 149 was used instead of the default timestamp. Ranked gene lists were tested against the oncogenic and hallmark MSigDb collections (v. 7.5.1).

### ChIP-sequencing

CWR22Rv1 cells were seeded at a density of 2.4 × 10^7^ cells per plate on 10-cm plates in RPMI 1640 with 10% FBS, allowed to adhere for 24 hours, and the medium was replaced with serum-free media for 9 hours. The medium was then replaced with serum-free medium containing 10 nM Prl (Biotechne, 682-PL) or refed with serum-free medium lacking Prl as a control for an additional 16 hours before cross-linking (1% formaldehyde for 10 min, quenched with glycine for 5 min). Nuclear pellets were sonicated on ice for eight cycles at 53% amplitude (each cycle: 10-s on/off pulse for 1-min total pulse duration, 10-min rest) using a SFX250 Sonifier (Branson). Lysates were immunoprecipitated with anti-Stat5ab antibody (Cell Signaling Technology, #D206Y) and A/G PLUS-Agarose (Santa Cruz Biotechnology, sc-2003) pre-blocked by tRNA (Sigma-Aldrich, #R8508). DNA was purified by a QIAquick PCR purification kit (Qiagen, 28106). For ChIP/next-generation sequencing (ChIP-seq), 5 ng of DNA (ChIP-enriched and input) was used for library creation with a ThruPLEX DNA-seq kit by UMGC. ChIP-seq libraries were sequenced at the UMGC using a NextSeq2000 2 × 50 bp (100 cycles) setting. FASTQ files were aligned to the GRCh38 version of the human genome using bwa aln (v 0.7.17). Only the first read was used in the alignment. Bedtools intersect (v 2.29.2) was used to remove aligned reads that overlapped with ENCODE’s exclude file downloaded from www.encodeproject.org/annotations/ENCSR636HFF. Bedtools genomecov (v 2.29.2) was used to convert the bam alignment file to a bedGraph file, and the bedGraphToBigWig program from UCSC (https://hgdownload.soe.ucsc.edu/admin/exe/linux.x86_64.v369/) was used to convert the file to a BigWig file. BigWIg files were loaded and coverage tracks were visualized in Integrative Genomics Viewer.

### Cell viability analysis

In the indicated experiments, fixed alive cells were stained with 0.5% crystal violet solution (MP Biomedicals), images were captured using a Bio-Rad Gel Doc XR System, and the fraction of surviving attached cells was counted (Bio-Rad) and analyzed by ImageJ.

### Ex vivo tumor explant cultures of patient-derived prostate cancers

PC specimens were obtained from patients undergoing radical prostatectomy (table S2) and were deidentified nondiagnostic excess tissue available for research purposes. The fresh human prostate specimens were obtained through the Medical College of Wisconsin Tissue Bank under institutionally approved IRB protocol (CPR00012448) from consented patients and in compliance with federal regulations governing research on deidentified specimens and/or clinical data [45 CFR 46102(f)]. Within 1 hour of surgery, the PC tissues were cultured following the procedures described previously (*33*, *35*, *36*, *47*, *51*, *52*). Briefly, PC tissue was cut into approximately 1-mm^3^ pieces in plain culture medium and transferred onto the culture matrix on grids in petri dishes. The basal medium was medium 199 with Earle’s salts (Sigma-Aldrich) containing 10% FBS, penicillin G (100 IU/ml), streptomycin sulfate (100 μl/ml), and glutamine (100 μg/ml), supplemented with insulin (0.08 IU/ml; Novo Nordisk) dexamethasone (100 nM; Sigma-Aldrich) and DHT (100 nM; Sigma-Aldrich). Explants (10 to 15 explants per treatment) were cultured for 7 days in the absence or presence of IST5-002 (12.5 μM), and the growth medium was changed every other day.

### Human prostate cancer xenograft tumor studies

Castrated male athymic nude mice (Taconic) were cared for according to the institutional guidelines and following the Institutional Animal Care and Use Committee–approved protocol. Following the procedures previously described in (*32*, *36*, *37*, *40*, *47*, *86*), mice were implanted with sustained release DHT pellets (60-day release, one pellet per mouse, Innovative Research of America) 7 days before PC cell inoculation. Briefly, 1.5 × 10^7^ CWR22Pc cells were mixed with 0.2 ml of Matrigel (BD Biosciences) and inoculated subcutaneously (sc) into flanks of nude mice (one tumor per mouse) as previously described (*32*, *33*). ENZ was dissolved in 0.5% Tween 80 (Sigma-Aldrich)/phosphate-buffered saline (PBS), and IST5-002 in 0.3% hydroxypropyl cellulose (Sigma-Aldrich)/H_2_O.

For the monotherapy study (Fig. 10C and fig. S7), mice (five mice per group) were treated daily for 32 days by oral gavage with vehicle (0.5% Tween 80/PBS) or ENZ (30 mg/kg), or by intraperitoneal (ip) injection with IST5-002 (50 mg/kg). DHT pellets were removed from the mice in the castration group concurrently with the start of the treatment period. Tumor dimensions were measured using Vernier calipers three times per week and tumor volumes were calculated using the following formula: (3.14 × length × width × depth)/6. Mice were euthanized, and the tumor tissues were harvested at the end of the 32-day treatment period, or before this endpoint if tumor sizes reached 15 to 20 mm in diameter, and tumor tissues were harvested. Tumor growth rates were calculated from the beginning of drug treatment and are presented as fold changes in the tumor volume of each group.

For the sequential therapy study (Fig. 10D and fig. S7, B and C), mice (10 mice per treatment group) were treated daily for 13 days (phase 1) by oral gavage with vehicle (0.5% Tween 80/PBS) or ENZ (30 mg/kg) as the first-line therapy. On day 13, mice were randomly distributed into the indicated second-line therapy groups and treated daily for an additional 18 days (phase 2) by oral gavage with vehicle, ENZ (30 mg/kg), or by intraperitoneal injection with IST5-002 (50 mg/kg). Tumor dimensions were measured twice per week and tumor volumes were calculated as described for the monotherapy study. Mice were euthanized when tumor sizes reached 15 to 20 mm in diameter in the vehicle-treated group (day 31), and the tumor tissues were harvested.

### Statistical analyses

Comparisons between groups of interest were performed using the analysis of variance (ANOVA) method with post hoc pairwise testing using two-sample *t* tests. Normality assumptions were evaluated using the Shapiro-Wilk test, while homoscedasticity was checked using Bartlett’s test. Wherever violations were observed, either with respect to the normality or homoscedasticity assumptions, the nonparametric Kruskal-Wallis test was used instead of post hoc testing using the Wilcoxon rank sum test. In all post hoc testing and multiple comparison scenarios, Bonferroni corrections were used to maintain overall type I error levels at or below 0.05. For all hypothesis tests, two-sided tests at the 0.05 level are considered (with multiplicity adjustments as indicated previously). All analysis was performed using R, version 4.2.2.
