# Supplementary material for: Stat5 induces androgen receptor (AR) gene transcription in prostate cancer and offers a druggable pathway to target AR signaling
Source: Sci Adv. 2024 Feb 28;10(9):eadi2742. doi: 10.1126/sciadv.adi2742 (PMC10901378; doi:10.1126/sciadv.adi2742)
Supplement: Supplementary file 1 — Figs. S1 to S7 Tables S1 and S2 Legends for data files S1 to S8 [file sciadv.adi2742_sm.pdf]

Science Advances
AAAS

Supplementary Materials for

Stat5 induces androgen receptor (*AR*) gene transcription in prostate cancer and offers a druggable pathway to target AR signaling

Cristina Maranto *et al.*

Corresponding author: Marja T. Nevalainen, marja.nevalainen@jefferson.edu

Sci. Adv. **10**, eadi2742
DOI: 10.1126/sciadv.adi2742

The PDF file includes:

Figs. S1 to S7
Tables S1 and S2
Legends for data files S1 to S8

Other Supplementary Material for this manuscript includes the following:

Data files S1 to S8

Supplementary Fig 1

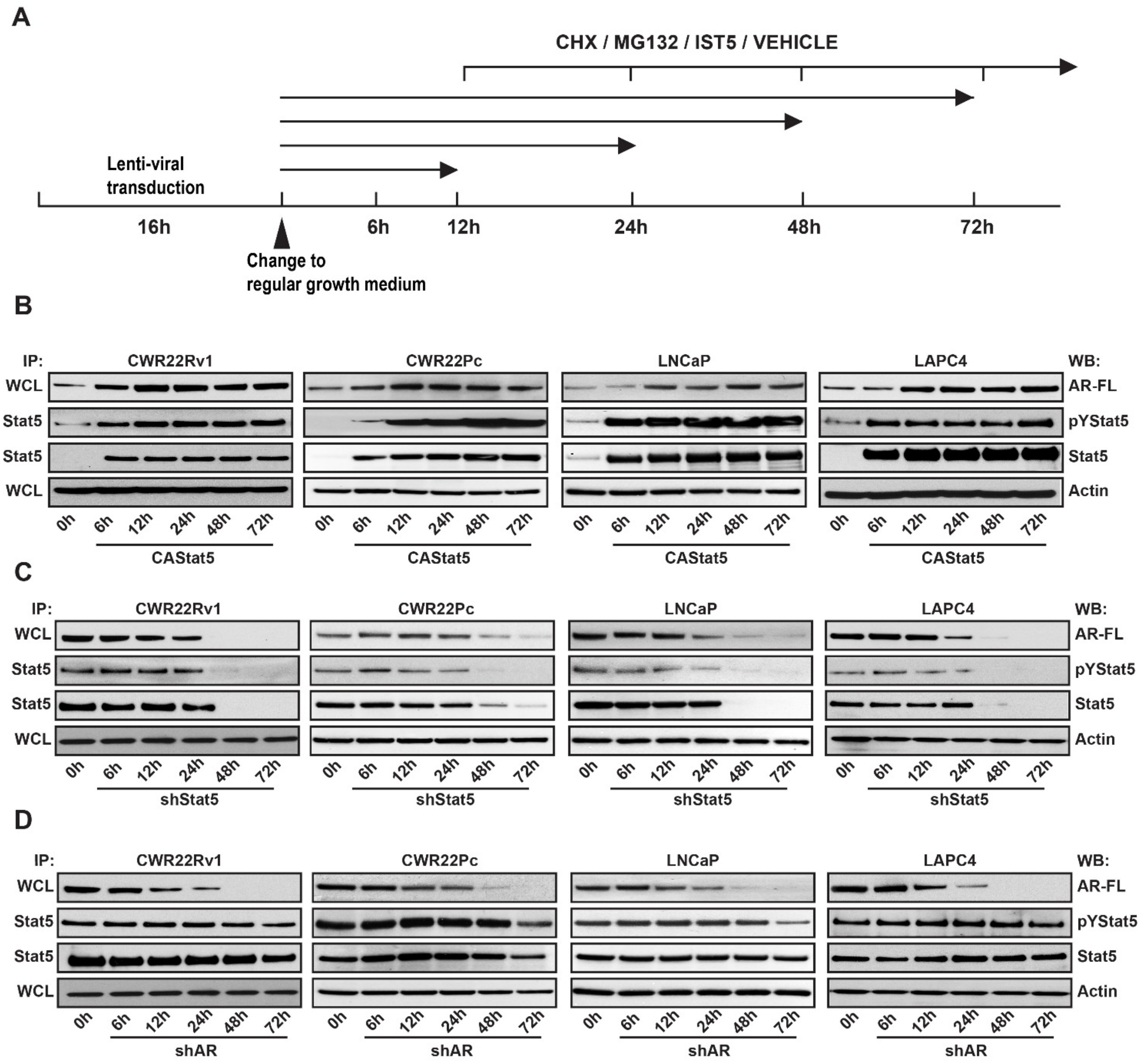

Supplementary Figure 1. Lentiviral transduction scheme of cells and t**imeline of Stat5-regulation of AR protein levels in prostate cancer cell lines. (A)** In all experiments, lentivirus containing culture media were changed to regular growth media 16 h after the initiation of lentiviral gene transduction. This time-point served as the start for counting the length of the lenti-viral gene expression. **(B)** CAStat5, **(C)** shStat5 or **(D)** shAR were lentivirally expressed in CWR22Rv1, CWR22Pc, LNCaP and LAPC 4 cells for 0, 6, 12, 24, 48 or 72 h, as indicated. Whole cell lysates (WCL) were western blotted (WB) for AR-FL and Actin. Stat5 was immunoprecipitated (IP) and blotted for phosphorylated active Stat5 (pStat5) and total Stat5.

Supplementary Fig 2

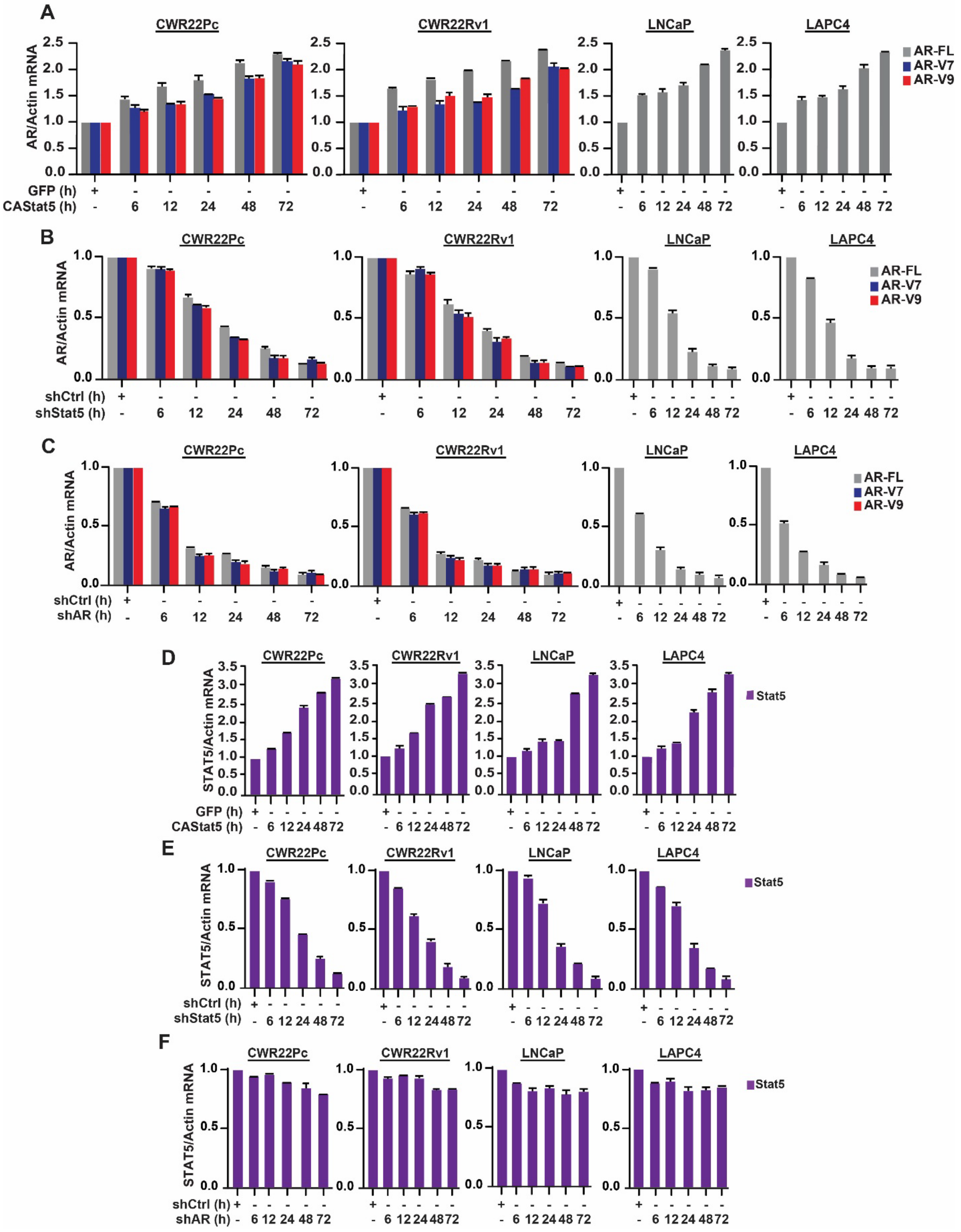

Supplementary Figure 2. Timeline of Stat5-regulation of AR-FL, AR-V7 and AR-V9 mRNA levels in prostate cancer cell lines. (A, D) CAStat5, **(B, E)** shStat5 or **(C, F)** shAR were lentivirally expressed in CWR22Pc, CWR22Rv1, LNCaP and LAPC 4 cells for 0, 6, 12, 24, 48 or 72 h, as indicated. AR-FL, AR-V7 and AR-V9 mRNA levels (**A, B and C**) and Stat5 mRNA levels (**D, E and F**) were determined by qRT-PCR.

Supplementary Fig 3

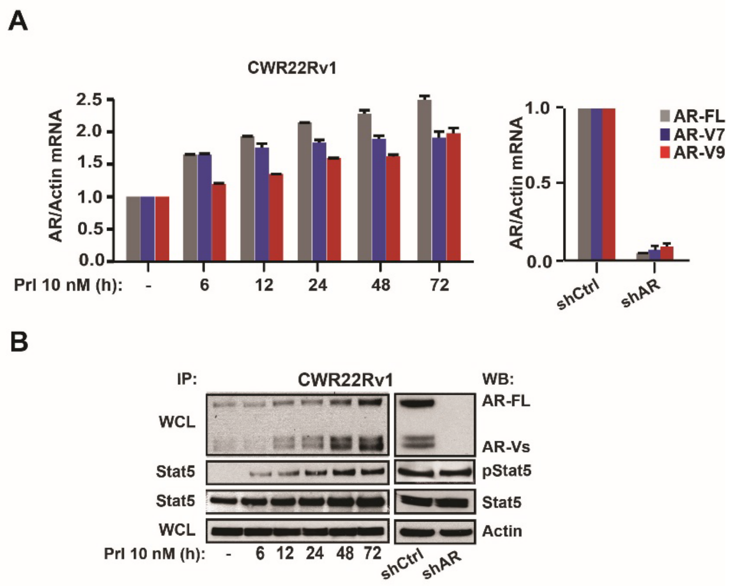

Supplementary Figure 3. Timeline of cytokine-induction of AR mRNA and protein levels in PC cells. CWR22Rv1 cells were treated with human prolactin (Prl) (10 nM) for the indicated periods of time. As comparison, shRNA targeting AR was lentivirally expressed in CWR22Rv1 cells for 72 h. AR-FL, AR-V7 and AR-V9 mRNA levels were determined by qRT-PCR. Cells from parallel wells were analyzed for protein levels of AR-FL and AR-Vs by western blotting (WB) of whole cell lysated (WCL) with actin as loading control. Stat5 was immunoprecipitated (IP:ed) and blotted for phosphorylated Stat5 (pStat5) and total Stat5.

Supplementary Fig 4

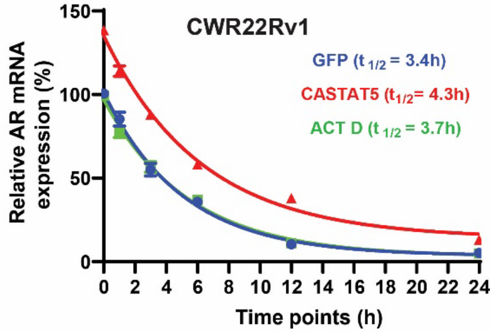

Supplementary Figure 4. Active Stat5 does not affect the stability of the AR mRNA in prostate cancer cells. CAStat5 or GFP were lentivirally expressed in CWR22Rv1 cells for 72 h followed by treatment of the cells with Actinomycin D (5 μM) with non-infected cells treated with Actinomycin D as an additional control. Cells were harvested in guanidinium thiocyanate buffer at the indicated time points and RNA was extracted and analyzed by qRT-PCR for AR-FL.

Supplementary Fig 5

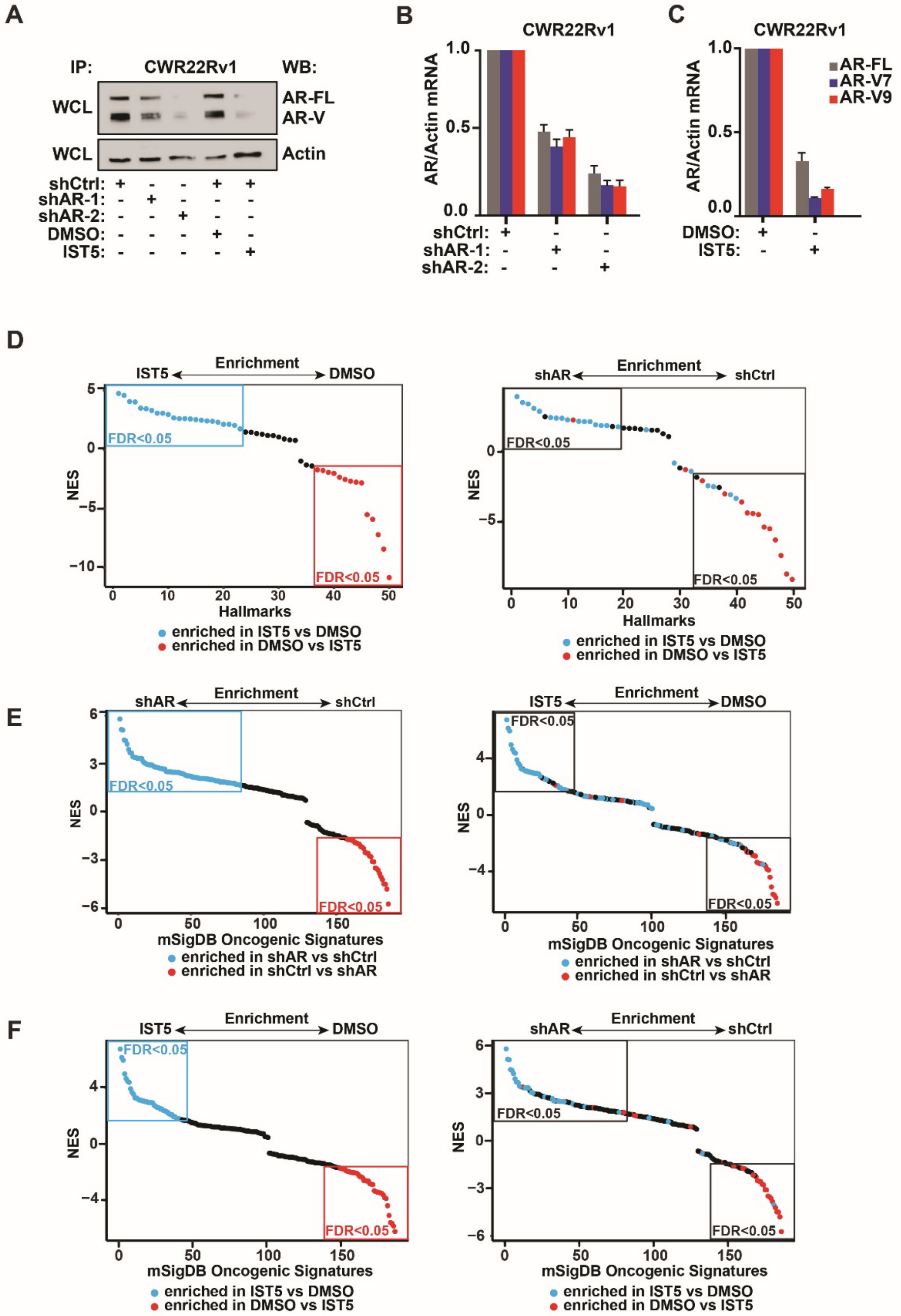

Supplementary Figure 5. IST5-002 (IST5) mediated inhibition of Stat5 causes similar effects to lentiviral knockdown of AR. (A) Western blot of AR-FL and AR-V protein expression in CWR22Rv1 cells infected with two independent shRNAs targeting AR (shAR-1 and shAR-2) or control shRNA (shCtrl) and treated with 800 nM IST5-002 (IST5) or vehicle control (DMSO). (**B, C**) mRNA levels of AR-FL, AR-V7, and AR-V9 measured using quantitative RT-PCR in CWR22Rv1 cells treated as in A. (**D)** Normalized Enrichment Scores (NES) for the 50 HALLMARK gene sets derived from gene set enrichment analysis (GSEA) in the CWR22Rv1 RNA-seq data reflecting Stat5 activity (IST5-002 vs DMSO, left) and AR activity (shAR vs shCtrl, right). Dots are colored blue or red based on whether they were positively or negatively enriched in IST5-002 vs DMSO with an FDR<0.05. (**E, F)** Normalized Enrichment Scores (NES) for the 189 mSigDB Oncogenic Signature gene sets derived from gene set enrichment analysis (GSEA) in the CWR22Rv1 RNA-seq data reflecting Stat5 activity (IST5-002 vs DMSO) and AR activity (shAR vs shCtrl). Dots are colored blue or red based on whether they were positively or negatively enriched in shAR vs shCtrl (**E**) or IST5-002 vs DMSO (**F**) with an FDR<0.05.

Supplementary Fig. 6

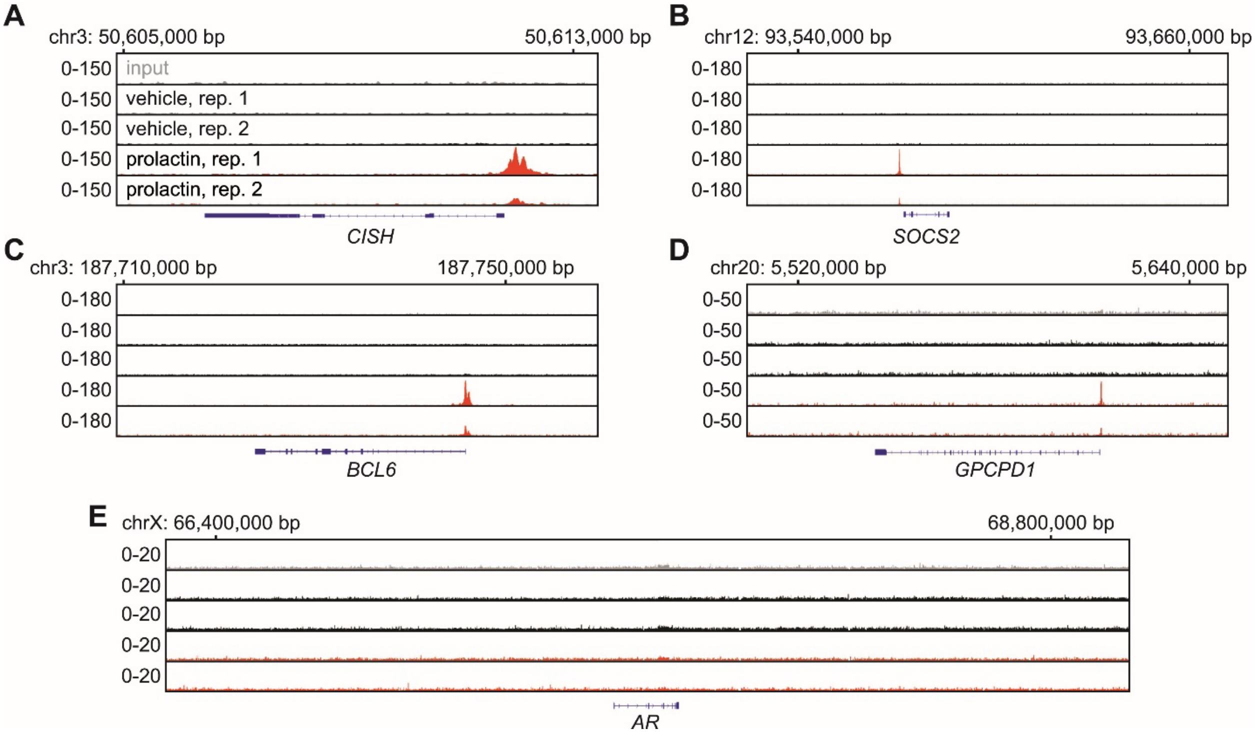

Supplementary Figure 6. The *AR* locus lacks evident binding of active Stat5. **(A-D)** Gene track view of Stat5 ChIP-seq data at the *CISH* **(A)**, *SOCS2* **(B)**, *BCL6* **(C)**, and *GPCPD1* **(D)** gene loci. Tracks are from input DNA (no antibody) generated from 22Rv1 cells (gray), or two independent biological replicates (rep. 1 and rep. 2) of DNA immunoprecipitated from 22Rv1 cells cultured in the presence (red) or absence (black) of 10 nM prolactin for 16 h. **(E)** Gene track view of STAT5 ChIP-seq data at the AR locus and its surrounding ~2 Mbp, which includes a known *AR* enhancer located approximately 650 kb centromeric to *AR*. Tracks are colored as in **(A)**.

Supplementary Fig 7

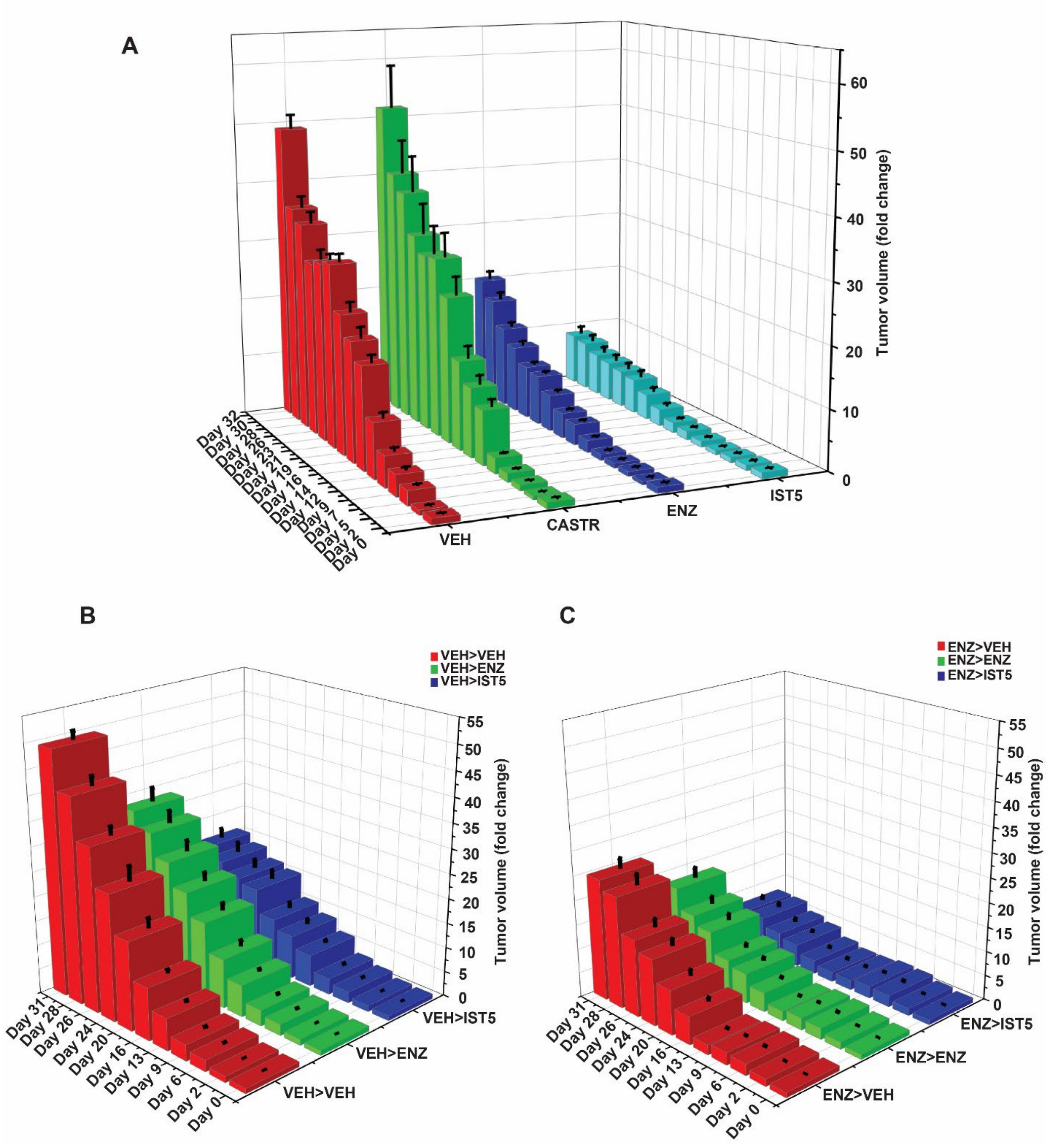

Supplementary Figure 7. IST5-002 (IST5) suppresses growth of both androgen-dependent and ENZ-resistant xenograft tumors in nude mice. (**A)** Inhibition of Stat5 by IST5-002 (IST5) suppressed androgen-sensitive CWR22Pc xenograft tumor growth more effectively than enzalutamide (ENZ), as shown by tumor growth curves. CWR22Pc cells were inoculated subcutaneously (s.c.) into flanks of castrated athymic nude mice supplied with sustained-release DHT pellets. Mice were surgically castrated or treated daily with vehicle, enzalutamide ENZ (30 mg/kg) or IST5-002 (50 mg/kg) for 32 days, and tumor growth rates were calculated for each treatment group and are presented as fold changes in tumor volume (volume at timepoint/volume at treatment start). (**B)** A two-phase *in vivo* experiment using vehicle or enzalutamide (ENZ) **(C)** as first-line therapy (phase I, 13 days) and vehicle, ENZ or IST5-002 as second-line therapy (phase II, 18 days). On day 31, mice were sacrificed and CWR22Pc xenograft tumors collected for analyses. Tumor dimensions were measured twice per week and tumor volumes calculated (*1*) as described in (**A)**.

Supplementary Table 1. Antibodies used in the study.

Antibody	Manufacturer	Dilution	Application	Catalog No.
Stat5a and Stat5b	Millipore	4:1000	IP	Customized
pY694 (Stat5)	BD Biosciences	1:1000	WB	611965
Stat5a/b	Cell Signaling		ChIP	D206Y
Stat5a/b	BD Biosciences	1:1000	WB	610192
AR	Biogenex	1:1000	WB	MU256-UC
PSA	Dako	1:5000	WB	A0562
Actin	Sigma Aldrich	1:4000	WB	A2066
Goat anti-mouse	BD Biosciences	1:2000	WB	554002
Goat anti-rabbit	BD Biosciences	1:2000	WB	554021

IP, immunoprecipitation; WB, Western blot; IFC, immunofluorescence cytochemistry; IHC, immunohistochemistry.

Supplementary Table 2. Patient-derived prostate cancers cultured *ex vivo* in tumor explant cultures

		**IST5-002-treated PCs (n=6) Median (range)**
Gleason score		**n (%)**
	4	0 (0)
	5	0 (0)
	6	0 (0)
	7	3 (50)
	8	1 (16.67)
	9	2 (33.33)
	10	0 (0)
Metastases detected		**n (%)**
	Yes	0 (0)
	No	6 (100%)
	Unknown	0 (0)

Supplementary Data 1: Genes higher in shAR vs shC (2141 genes).xlsx

- genes up-regulated in CWR22Rv1 cells infected with shAR compared to shC

- represented by blue dots in Fig. 5D, left panel

Supplementary Data 2: Genes lower in shAR vs shC (2201 genes).xlsx

- genes down-regulated in CWR22Rv1 cells infected with shAR compared to shC

- represented by red dots in Fig. 5D, left panel

Supplementary Data 3: Genes higher in IST5 vs DMSO (7068 genes).xlsx

- genes up-regulated in CWR22Rv1 cells treated with IST5-002 compared to DMSO

-represented by blue dots in Fig. 5D, right panel

Supplementary Data 4: Genes lower in IST5 vs DMSO (7040 genes).xlsx

- genes down-regulated in CWR22Rv1 cells treated with IST5-002 compared to DMSO

- represented by red dots in Fig. 5D, right panel

Supplementary Data 5: Gsea_report_shARvsshC_pos.xlsx

- hallmark and oncogenic gene sets in the molecular signatures database (mSigDB) that are positively enriched in CWR22Rv1 cells infected with shAR compared with shC

- represented by top left FDR<0.05 quadrant in Fig. 5E, top panel (includes HALLMARK_APOPTOSIS)

- the blue dots throughout Fig. 5E

Supplementary Data 6: Gsea_report_shARvsshC_neg.xlsx

- hallmark and oncogenic gene sets in the molecular signatures database (mSigDB) that are negatively enriched in CWR22Rv1 cells infected with shAR compared with shC

- represented by bottom right FDR<0.05 quadrant in Fig. 5E, top panel (includes HALLMARK_E2F_TARGETS)

- the red dots throughout Fig. 5E

Supplementary Data 7: Gsea_report_IST5vsDMSO_pos.xlsx

- hallmark and oncogenic gene sets in the molecular signatures database (mSigDB) that are positively enriched in CWR22Rv1 cells treated with IST5-002 compared with DMSO

- represented by top left FDR<0.05 quadrant in Fig. 5E, bottom panel (includes HALLMARK_APOPTOSIS)

Supplementary Data 8: Gsea_report_IST5vsDMSO_neg.xlsx

- hallmark and oncogenic gene sets in the molecular signatures database (mSigDB) that are negatively enriched in CWR22Rv1 cells treated with IST5-002 compared with DMSO

- represented by bottom right FDR<0.05 quadrant in Fig. 5E, bottom panel (includes HALLMARK_E2F_TARGETS)
